# Repeated sleep deprivation decreases the flux into hexosamine biosynthetic pathway/*O*-GlcNAc cycling and aggravates Alzheimer’s disease neuropathology in adult zebrafish

**DOI:** 10.1186/s12974-023-02944-1

**Published:** 2023-11-09

**Authors:** Jiwon Park, Dong Yeol Kim, Geum-Sook Hwang, Inn-Oc Han

**Affiliations:** 1 Department of Biomedical Science, Program in Biomedical Science and Engineering, College of Medicine, Inha University, Incheon, Korea; 2 Integrated Metabolomics Research Group, Western Seoul Center, Korea Basic Science Institute, Seoul, Korea; 3 College of Pharmacy, Chung-Ang University, Seoul, Korea; 4 Department of Physiology and Biophysics, College of Medicine, Inha University, 100 Inha Ro, Nam-Gu, Incheon, 22212 Korea

**Keywords:** Sleep, Hexosamine, Alzheimer’s disease, Zebrafish

## Abstract

This study investigated chronic and repeated sleep deprivation (RSD)-induced neuronal changes in hexosamine biosynthetic pathway/*O*-linked N-acetylglucosamine (HBP/*O*-GlcNAc) cycling of glucose metabolism and further explored the role of altered *O*-GlcNAc cycling in promoting neurodegeneration using an adult zebrafish model. RSD-triggered degenerative changes in the brain led to impairment of memory, neuroinflammation and amyloid beta (Aβ) accumulation. Metabolite profiling of RSD zebrafish brain revealed a significant decrease in glucose, indicating a potential association between RSD-induced neurodegeneration and dysregulated glucose metabolism. While RSD had no impact on overall *O*-GlcNAcylation levels in the hippocampus region, changes were observed in two *O*-GlcNAcylation-regulating enzymes, specifically, a decrease in *O*-GlcNAc transferase (OGT) and an increase in *O*-GlcNAcase (OGA). Glucosamine (GlcN) treatment induced an increase in *O*-GlcNAcylation and recovery of the OGT level that was decreased in the RSD group. In addition, GlcN reversed cognitive impairment by RSD. GlcN reduced neuroinflammation and attenuated Aβ accumulation induced by RSD. Repeated treatment of zebrafish with diazo-5-oxo-l-norleucine (DON), an inhibitor of HBP metabolism, resulted in cognitive dysfunction, neuroinflammation and Aβ accumulation, similar to the effects of RSD. The pathological changes induced by DON were restored to normal upon treatment with GlcN. Both the SD and DON-treated groups exhibited a common decrease in glutamate and γ-aminobutyric acid compared to the control group. Overexpression of OGT in zebrafish brain rescued RSD-induced neuronal dysfunction and neurodegeneration. RSD induced a decrease in *O*-GlcNAcylation of amyloid precursor protein and increase in β-secretase activity, which were reversed by GlcN treatment. Based on the collective findings, we propose that dysregulation of HBP and *O*-GlcNAc cycling in brain plays a crucial role in RSD-mediated progression of neurodegeneration and Alzheimer’s disease pathogenesis. Targeting of this pathway may, therefore, offer an effective regulatory approach for treatment of sleep-associated neurodegenerative disorders.

**Supplementary Information:**

The online version contains supplementary material available at 10.1186/s12974-023-02944-1.

## Introduction

Sleep is an essential physiological process that plays a crucial role in maintaining multiple biological functions, including metabolic homeostasis, energy replenishment, synaptic formation associated with learning, and reactivation of neurons [1, 2]. Depending on the duration of sleeplessness, sleep deprivation (SD) can be categorized as either acute or chronic. Acute SD is defined as continuous sleep deprivation lasting for several hours to days, while chronic SD refers to repeated or fragmented sleep deprivation over the course of several weeks or months [3]. Emerging evidence has linked both acute and chronic SD to cognitive dysfunction [4–6]. Disturbed sleep is often observed in patients with various neurodegenerative and neuropsychiatric disorders [7–9]. Prolonged and repeated SD (RSD) is particularly associated with impaired neural function, leading to neurodegenerative disorders, such as Parkinson’s disease and Alzheimer’s disease (AD) [10]. While the mechanisms underlying the initiation or progression of neurodegenerative diseases following chronic SD have not been clarified, some of the effects of chronic SD are potentially attributable to an increase in neuroinflammation and altered brain energy metabolism [11–13].

Brain energy metabolism is intricately linked to neuronal activity, ensuring that energy supply is appropriately adjusted to neuronal needs. SD triggers substantial reorganization of regional cerebral metabolic activity. For example, SD has been shown to induce a ~ 10–20% decrease in glucose uptake across all brain regions in both humans and rodents [14–16]. Glucose serves as the primary energy source in brain and is a vital precursor for synthesis of downstream metabolites through various pathways, including glycolysis and hexosamine biosynthetic pathway (HBP). HBP is a fundamental metabolic process that integrates multiple essential nutrient pathways, including glucose, lipid, nucleotide, and amino acid metabolism [17]. Approximately 2–5% of glucose is metabolized in HBP, leading to the production of hexosamine derivatives [18]. The end-product of HBP, UDP-N-acetylglucosamine (UDP–GlcNAc), serves as a substrate for protein *O*-GlcNAc post-translational modification (*O*-GlcNAcylation). *O*-GlcNAcylation refers to a widespread and regulatory protein modification in which the addition of GlcNAc moieties to serine or threonine residues of target proteins is catalyzed by the enzyme *O*-GlcNAc transferase (OGT) [19]. Conversely, removal of these GlcNAc moieties is catalyzed by the enzyme *O*-GlcNAcase (OGA) [19]. This dynamic interplay between OGT and OGA controls the *O*-GlcNAcylation status of proteins, thereby modulating various physiological processes, including cellular signaling, gene expression, protein stability, and other important pathways [20]. Emerging evidence presents compelling support for a correlation between dysregulated brain *O*-GlcNAc levels and various neurodegenerative conditions, including neuronal cell death, neuroinflammation, increased production of hyperphosphorylated tau and amyloidogenic Aβ peptides, and memory deficits [21]. While the issue of whether *O*-GlcNAcylation plays a protective role or exacerbates brain degeneration is currently a matter of debate, *O*-GlcNAc appears to protect against neurodegeneration overall and its downregulation may, therefore, be detrimental to normal brain function. *O*-GlcNAcylation is decreased in brain tissue of AD patients compared to control tissue and expression of OGT protein is reduced in the cortical region of AD brain [22]. Pharmacological upregulation of *O*-GlcNAc is currently under investigation as a potential therapeutic strategy for AD and other neurodegenerative diseases.

AD is characterized by the deposition of amyloid‐β (Aβ), increased tau phosphorylation, and neuronal loss in the brain. Sleep disorders are commonly observed in patients with AD and tend to worsen with disease progression [23]. Memory decline in AD may be a result of impaired sleep-dependent memory consolidation, as sleep modulates synaptic connections in the hippocampus that play an important role in learning and memory [24]. Studies on both mice and humans have shown increased accumulation of Aβ in the brain and cerebrospinal fluid of individuals with sleep disorders [25, 26], suggesting exacerbation of the pathological progression of AD. Our current knowledge of the molecular pathways through which SD accelerates memory dysfunction or AD progression is limited. Considerable efforts are underway to elucidate these underlying mechanisms, with the aim of identifying potential therapeutic targets.

In the current study, adult zebrafish were utilized as a novel experimental model for chronic RSD. This model involved three consecutive days of SD followed by 4 days of recovery over five repeated cycles. The diverse functional and molecular changes in the brain induced by RSD in our model were examined over a span of 5 weeks. The molecular mechanisms underlying cognitive impairment and neurodegeneration induced by RSD were further explored. We specifically focused on alterations in the brain glucose metabolism pathways, particularly HBP, and the role of *O*-GlcNAc cycling in SD-induced brain degeneration and AD pathogenesis.

## Materials and methods

### Chemicals and reagents

Reagents were purchased from Sigma Chemical (MO, USA) or Tocris (Bristol, UK), otherwise noted.

### Zebrafish handling

Adult zebrafish (Danio rerio, 3–6 months) were obtained from Fishzzang (Jinchun, Korea) and were used for the experiments. All studies were procedure in accordance with the guidelines of the Institutional Animal Care and Use Committee of the Inha University in Incheon (INHA 220719-832). The fish were kept in controlled illumination (12 h light/12 h dark cycle), temperature (26 ± 1 °C), and pH (6.5–7.5) with supplied air bubbles. Water was filtered through a multi-stage filtration system. The fish were fed flake food (Terabits; Luzerne, Singapore) twice a day using an automated fish feeder (AF012; DoPhin, London, UK). All of experiments and measurements were performed with both sexes (~ 50:50 male:female ratio) in a blinded and randomized manner.

### Repeated sleep deprivation of adult zebrafish

All zebrafish were acclimated to the laboratory environment for 7 days prior to the induction of sleep deprivation (SD). In this study, extended light exposure was used to establish the SD model. Altering exposure to ambient light is the most effective way to modulate sleep duration in zebrafish [27, 28]. It has also been indicated that sleep deprivation through light exposure does not significantly affect stress responses in zebrafish. Adult zebrafish were placed in a rectangular box with dimensions of 50 cm × 50 cm × 50 cm in a controlled temperature environment (26 ± 1 °C). The box provided a controlled setting, where light conditions could be regulated automatically. Zebrafish were divided into four groups: a control group, a SD group for 3 days, an SD group followed by 4 days of sleep recovery (SD(R)) under a normal light–dark cycle, and a group subjected to five cycles of repeated SD(R) over a period of 5 weeks (RSD).

### Drug administration

Glucosamine (GlcN, 20 μg/g) was intraperitoneally injected on the day of every SD cycle induction during the RSD period using a Hamilton syringe (Hamilton, NV, USA). For chronic diazo-5-oxo-l-norleucine (DON) treatment, DON (500 ng/g) was intraperitoneally injected for five cycles, with each cycle comprising 3 days of injection followed by 4 days of recovery (DON (5X)). In certain experimental groups, an additional intraperitoneal injection of glucose (Glc, 20 μg/g) or GlcN (20 μg/g) was administered on the same day as the DON injection. After the final cycle of DON injections, either the fear context L/M test or brain analysis was conducted. For all injection experiments, an equal volume of PBS, matching the volume of the drug, was administered intraperitoneally.

### Body mass index (BMI) and blood glucose-level measurement

The height and weight of zebrafish were measured after anesthetization. The method for zebrafish blood collection was described [29]. Briefly, the tails of zebrafish were cut with scissors, and blood was collected. The collected blood samples were immediately analyzed twice using a glucose meter (OSANG Healthcare, Kyeonggi, Korea).

### AAV–OGT injection

The adeno-associated virus serotype 8 (AAV8) vector containing mouse OGT (mOGT), AAV8–CMV–mOGT–Myc–Flag (vOGT), was generated and purified by the Korea Institute of Science and Technology (KIST) Virus Facility (KIST, Seoul, Korea). AAV8 was engineered using DNA family shuffling technology, resulting in a hybrid capsid derived from eight standard AAV serotypes. The plasmids used for AAV vector production included the AAV8 serotype construct, the AAV transfer plasmid, a plasmid encoding rep and serotype-specific capsid proteins, and a plasmid encoding adenoviral helper sequences. The viral vectors were pseudotyped, with mOGT flanked by inverted terminal repeats of AAV8, and encapsulated within an AAV8 capsid. Adult zebrafish received intracranial injections of 2–3 μl of vOGT, which contained approximately 1 × 10^11^ genome copies per milliliter. These injections were administered into the inner part of the skull using a Hamilton syringe. After a 2-week period following the viral injection, the procedures for SD or RSD were initiated.

### Behavioral tests of zebrafish

During the tests, both the test room and the water were maintained at a temperature of 26 ± 1 °C. The behavioral tests were carried out between 9 a.m. and 7 p.m. To ensure stability and acclimation, zebrafish were placed in the test room at least 1 h before the start of each behavioral test.

1.1.1. Novel tank test

The novel tank test was performed to access anxiety-like behaviors in the adult zebrafish [30]. The novel tank used in the experiment was designed to be narrow and had different size and shape (20 cm × 5 cm × 15 cm) compared to the home tank. It was made of clear acrylic material. After a 5-min stabilization period in the test tank, the swimming patterns of the zebrafish were recorded for an additional 5 min using a CCD camera (JYCOS, Incheon, Korea). The recorded videos were later analyzed using ToxTrack software (https://toxtrac.sourceforge.io) [31]. The speed, swimming distance, mobility, freezing patterns, and preference zone of the zebrafish in the tank were measured.

2.2.2. Fear context learning and memory (L/M) test

The fear context L/M test evaluates how zebrafish associate a specific context, such as a light space, with fear-inducing stimuli like a dropping stone. It assesses their ability to remember and retain this association, as previously described [32].

3.3.3. T-maze test

The T-maze test was modified from the shoal preference test to assess an animal’s spatial perception and decision-making skills while taking into account their social preferences and measuring their memory function [33]. The T-maze included one long arm (starting zone) and two short arms (empty zone and friend zone), each separated by acrylic partitions.

4.4.4. Actogram test

To assess the sleep and wake patterns of the zebrafish, we conducted an actogram test. The actogram apparatus was designed with an infrared sensor system (Omron, Kyoto, Japan). This infrared sensor was positioned beneath the water surface, approximately 5 cm below, on one side of the testing tanks. The sensor data were recorded on a connected computer and subsequently analyzed using customized software developed by Scitechkorea (Seoul, Korea).

### Metabolites extractions of brain tissues and Gas Chromatography Mass spectrometry (GC–MS)/MS analysis for metabolites

The extraction and analysis method for glucose metabolites in the zebrafish brains was previously described [34]. In brief, the brains of adult zebrafish (*n* = 5/sample) were homogenized in 70% MeOH in water, and then subjected to chloroform (CHCl_3_) extraction. After centrifugation, the supernatant was evaporated under a stream of nitrogen for further analysis. For quantitative analysis of metabolites, gas chromatograph/tandem mass spectrometer analysis was performed on an Agilent 7890B gas chromatograph, equipped with a 7010-mass selective detector triple quadrupole (TQ) mass spectrometer system (Palo Alto, CA, USA). Chromatographic separation was achieved using a DB-5MS UI column (30 m × 0.25 mm I. D; 0.25 µm film thickness) from J&W Scientific (Santa Clara, CA, USA) capillary column.

### Neurotransmitter analysis by liquid chromatography mass spectrometry (LC–MS/MS)

The extraction and analysis method for serotonin, glutamate, acetylcholine (ACh), and γ-aminobutyric acid (GABA) in zebrafish brains followed a previously described protocol [32] using the Agilent 1290 Infinity LC and Agilent 6490 Triple Quadrupole MS system (Agilent Corp., CA, USA) in Korea Basic Science Research Institute. Serotonin-D4, glutamate-13C5, choline-D9, and GABA-D6 were used as internal standards. All samples were repeated three times. MS/MS analysis was performed in a positive ion mode.

### Immunofluorescence staining

For immunofluorescence staining, whole zebrafish brains were collected on ice after euthanization using ethyl 3-aminobenzoate methanesulfonate (MS-222). The tissue was then promptly fixed in 4% formaldehyde (Samchun, Seoul, Korea) at 4 °C for 2 h. The tissue was then washed with PBS and cryoprotected in 30% sucrose at 4 °C (for at least 12 h). Each brain was embedded in O.C.T compound (Sakura Finetek, Koto, Japan) in a tissue mold on dry ice. The frozen tissues were stored at − 80 °C and stabilized at − s20 °C for 1 h prior to sectioning. The brain was coronally sectioned into 6 μm thick slices using a cryostat (CM3050; Leica, Wetzlar, Germany) and mounted onto coated slides (Matsunami glass Ind., Ltd., Osaka, Japan). For staining, the slides were subjected to antigen retrieval by boiling in 10 mM citrate buffer (pH 6.0) for 5 min. The slides were rinsed with PBS-T (0.1% Triton X-100 in PBS) and blocked with 10% normal goat serum (Jackson ImmunoResearch, PA, USA) for 1 h. The sections were incubated with primary antibodies specific for *O*-GlcNAc (RL-2), IκBα, p65, OGT, Bace-1 (Santa Cruz, TX, USA), OGA (Proteintech, IL, USA), S100β (Vector laboratories, CA, USA), pTau, Aβ, APP (Cell Signaling Technology, MA, USA), GFAP, and Flag (Sigma). The signal was detected using IgG cross-adsorbed Alexa Flour 488 or 568 secondary antibodies (Invitrogen, CA, USA). The nuclei were counterstained with DAPI. The slides were mounted using Fluoromount-G Mounting medium (SouthernBiotech, AL, USA) and examined under a confocal LSM 510 META microscope (Carl Zeiss, Jena, Germany) at 40 × magnification. The fluorescent images were analyzed using ZEN 2009 Light Edition software. The quantification of the images was performed using the ImageJ program (NIH, MD, USA). The images were prepared with three tissue sections per zebrafish per slide.

### Reverse transcription and quantitative real-time PCR (qRT-PCR)

Total RNA from the zebrafish brain tissues (*n* = 3/sample) was isolated using TRizol™ (Invitrogen). The extraction of the RNA following the manufacturer’s protocols. Total RNA, reaction buffer, RNase inhibitor (Invitrogen), and GoScript™ Reverse Transcriptase (Promega, WI, USA) were mixed and incubated for an hour at 37 °C for reverse transcription. Real-time PCR was performed with a SYBR Green master mix (BioFact, Daejeon, Korea) with primers, and amplified by CFX Connect™ (BIO-RAD, CA, USA). The following primers (Macrogen, Seoul, Korea) were utilized in this study: *β-actin* forward, 5′- GTGCCCATCTACGAGGGTTA-3′, *β-actin* reverse, 5′-TCTCAGCTGTGGTGGTGAAG-3′, *glut1* forward, 5′-TAACGCTCCACAGAAGATCA-3′, *glut1* reverse, 5′-GGGCAATTTCTCCAACATAC-3′, *glut3* forward, 5′-GCTCCTCCTTCGAGATGATA-3′, *glut3* reverse, 5′-CTTCCCCACATCCTCATAAC-3′, *ogt* forward, 5′-AGCCATTGACACATACCGTC-3′, *ogt* reverse, 5′-CTTGAAGCTTTCCTTGCTGC-3′, *oga* forward, 5′-GCTTGAGGATGAGGAAGGTG-3′, *oga* reverse, 5′-AGCACCATGTGAGCCATTAG-3′, *appα* forward, 5′-GGTGGAGGTGCCGTCAGA-3′, *appα* reverse, 5′-CGCTCTGGATGTTGATGTGC-3′, *appβ* forward, 5′-TGACGGTGAAGATGATGAAG-3′, *appβ* reverse, 5′-CTTATCAGCACGAGGAAGGT-3′, *bace-1* forward, 5′-TTACATAGAGATGGCGGTGGG-3′, *bace-1* reverse, 5′-GAGGAGAGTGAGCGGTGGTAATA-3′, *psen-1* forward, 5′-AACCTCCTCCTGCTCTTCTT-3′, *psen-1* reverse, 5′-CATATTGAAGAGCCACACCA-3′, *psen-2* forward, 5′-GATTCTATCCTCGCTGATGC-3′, *psen-2* reverse, 5′-CAGACCATGGCAGATGAATA-3′. The results obtained for each target gene were normalized with the β-actin levels using the delta–delta Ct method. All experiments were replicated three independent times.

### Cell lysates preparation and western blot

Zebrafish whole brains (*n* = 3/sample) were harvested and homogenized in the RIPA buffer (50 mM Tris-pH 7.4, 150 mM NaCl, 0.25% deoxycholic acid, 1% NP-40, 1 mM EDTA–pH8.0) containing proteases, phosphatases and *O*-GlcNAcase inhibitors (1 mM PMSF, 1 mM DTT, 1 mg/mL aprotinin, 1 mg/mL leupeptin, 1 mM sodium orthovanadate, 1 mM sodium fluoride, 1 mM streptozotocin). The lysates were centrifuged, and the supernatants were transferred to new tubes. The samples were subjected to analysis by Western blotting. The separated proteins were probed with antibodies specific for *O*-GlcNAc (RL-2), OGT, β-actin (Santa Cruz) OGA (Proteintech), and APP (Cell Signaling Technology). The primary signals were detected by horseradish peroxidase (HRP)–secondary IgG antibodies (Abcam, Cambridge, UK), and HRP-bound proteins on the membranes were visualized using Enhanced Chemiluminescence (ECL, Amersham Biosciences, IL, USA). The protein bands were densitometric quantification with Image J program.

### WGA-pull down assay

Whole cell lysates of zebrafish brain (*n* = 3/sample) were prepared, and 500 μg of each sample was used for wheat germ agglutinin (WGA)-pull down assay. The proteins were incubated with pre-cleared WGA agarose beads (Vector laboratories, CA, USA) for 2 h at 4 °C on a rotator. After incubation, the agarose-conjugated proteins were washed with PBS. The agarose-bound proteins were eluted from the agarose by boiling with 5X SDS-PAGE sample buffer (Tech & Innovation, Chuncheon, Korea) then subjected to SDS–PAGE gel electrophoresis and analyzed using a Western blot.

### β-Secretase activity assay

To measure β-secretase activity, zebrafish brains were sacrificed, and the enzyme activity was measured using a kit (MAK237; Sigma) according to the manufacturer’s protocol. In brief, the brain sample was homogenized in the extraction buffer and then centrifuged. The resulting supernatant was used for analysis. For the analysis, an equal volume of the sample and reaction buffer were added to the wells of a 96-well plate. The plate was then placed in a hybridization machine and pre-incubated at 37 °C for 20 min. After the pre-incubation, the samples were incubated with an enzyme substrate for 1 h. The fluorescence signals were measured using a microplate reader (S1LFA; Biotek, VT, USA).

### Statistical analysis

All the graphs were generated and statistically analyzed using Prism software (version 8; GraphPad Software, CA, USA). The results were presented as means ± SEM (standard error of the mean). To determine significant differences between two groups, the Mann–Whitney test was employed. For comparisons among more than two groups, the Kruskal–Wallis test with the original false discovery rate (FDR) correction method of Benjamini and Hochberg was utilized. For GC–MS/MS data analysis, Mann–Whitney *U* test was utilized, and for LC–MS/MS data analysis, one-way ANOVA with post hoc Tukey’s multiple comparison tests were performed. Behavioral data analysis involved performing a two-way ANOVA with Tukey’s multiple comparisons test to assess differences between groups. For within-group comparisons, the Friedman test with ANOVA was performed. Statistical significance was defined as *p* < 0.05.

## Results

### Repeated sleep deprivation triggers L/M impairment, neuroinflammation and Aβ accumulation in brain of adult zebrafish

**Fig. 1:**
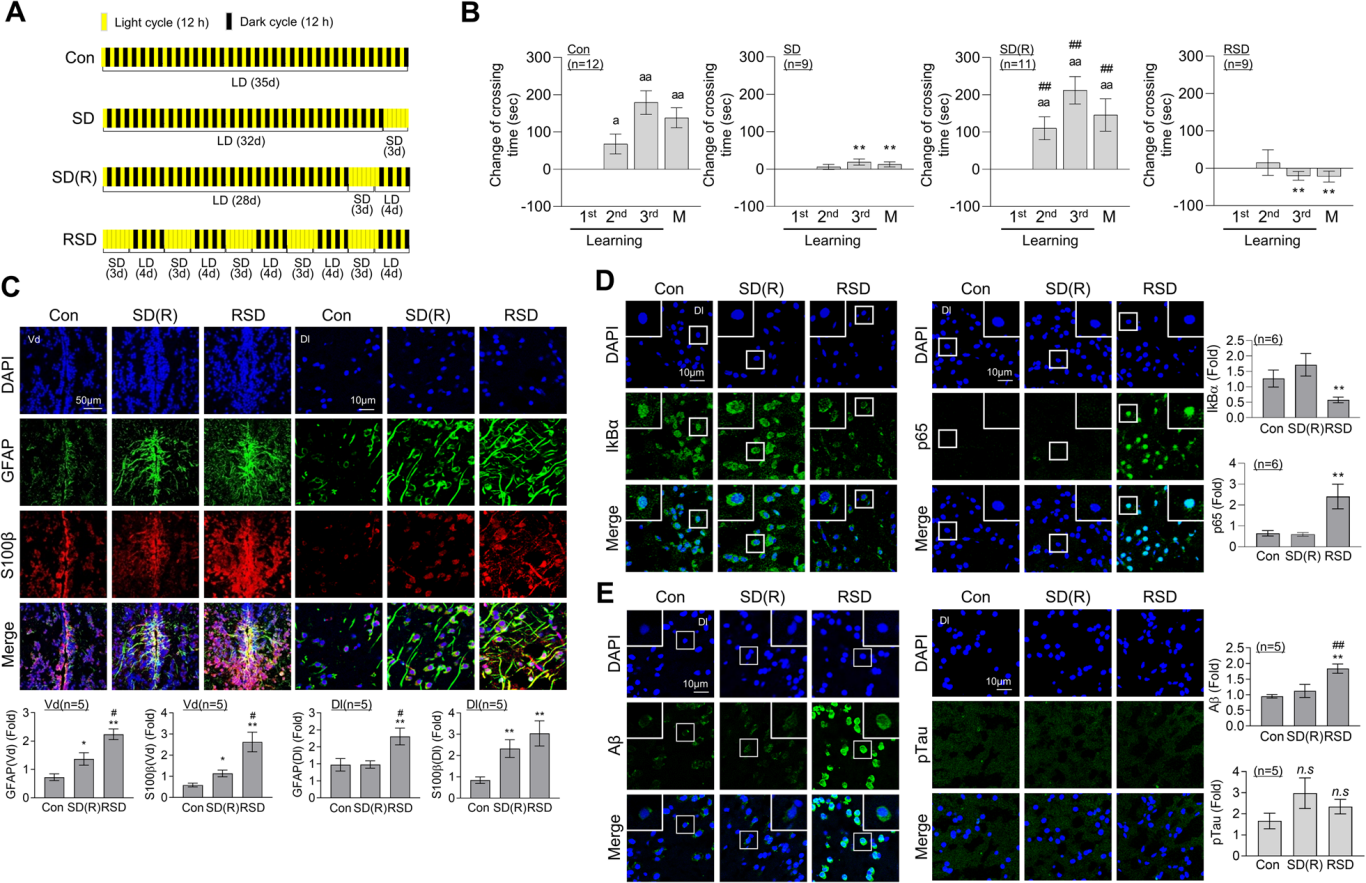
SD- or RSD-induced L/M deficits, neuroinflammation, and Aβ accumulation in the brain of adult zebrafish. For sleep deprivation (SD), zebrafish were exposed to extended light for 72 h (3 days). After SD, the fish had a 4-day recovery period with a normal sleep–wake pattern (SD (R)). Repetitive sleep deprivation (RSD) involved five repeated cycles of SD (R). **A** Experimental schematic illustration of SD, SD (R), and RSD. **B** Graphs represent the contextual L/M test, showing altered crossing times compared to those observed during the first learning session. Values represent the mean ± SEM (*n* = 9–11/group). For statistical analysis, a within-group comparison was conducted using the Friedman ANOVA test with the original Benjamini and Hochberg false discovery rate (FDR). (^a^*p* < 0.05, ^aa^*p* < 0.01 versus 1st learning). A two-way ANOVA with Tukey’s multiple comparisons test was performed for between-group comparisons (*p < 0.05, **p < 0.01 versus Con, ^##^*p* < 0.01 versus SD). **C**–**E** Representative confocal images (× 40) of DAPI (blue), GFAP (**C**, green), S100β (**C**, red), IκBα (**D**, left, green), p65 (**D**, right, green), Aβ (**E**, left, green), pTau (**E**, right, green), and merged immunofluorescence staining are shown for the dorsal nucleus of the Vd or Dl region of the telencephalic area in zebrafish brain. Enlarged images are presented within white boxes. The graphs display the quantitative results for each antibody with normalization based on DAPI levels (*n* = 5 ~ 6/group). For statistical analysis, the Kruskal–Wallis test with the original Benjamini and Hochberg FDR was conducted (*p < 0.05, **p < 0.01 versus Con, ^#^*p* < 0.05, ^##^*p* < 0.01 versus SD(R))

We established a zebrafish animal model of chronic and repeated sleep deprivation (RSD) lasting for 5 weeks. SD was induced for three consecutive days by extended light exposure, followed by a recovery period of 4 days (SD(R)) under a normal light–dark cycle. This cycle of SD(R) was repeated five times over a span of 5 weeks to create a model of chronic RSD (Fig. 1A). The sleep–wake cycle of zebrafish during SD induction was assessed using an actogram. The control group exhibited significantly higher activity levels during the daytime compared to nighttime. In contrast, the sleep-deprived zebrafish exposed to extended light showed similar activity percentages during both the day and night, indicating disrupted sleep patterns during the RSD period (Additional file 1**:** Fig. S1A). We subsequently assessed the impairment in memory function induced by RSD. Consistent with our previous findings [27], a single episode of SD led to significant impairment of fear-context learning and memory (L/M) function in zebrafish, which was almost completely restored to normal levels over the 4-day recovery period following SD (Fig. 1B). However, in our RSD model, sustained impairment of L/M function occurred even after the recovery period. Spatial perception abilities and social preference functions were further examined using a modified T-maze test. SD and RSD did not induce significant changes relative to the control group in terms of spatial function, motility, and social preference (Additional file 1**:** Fig. S1B). Novel tank tests (NTT), including swimming exploration patterns, erratic movements, and freezing [32], were conducted to further assess anxiety levels. No significant differences were observed in preferred exploration area, exploration distance, swimming speed, swimming rate, and freezing patterns of zebrafish between control and SD or RSD groups (Additional file 1**:** Fig. S1C, D). The collective data suggest that both SD and RSD promote impairment of fear-motivated L/M but do not impact social behavior, spatial perception, or anxiety-like behavior in adult zebrafish.

Next, we investigated whether SD and RSD induce changes in neuroinflammation. Expression of the astrocyte activation marker, glial fibrillary acidic protein (GFAP), was increased in both the ventral (Vd) and dorsal lateral (Dl) zones of the telencephalic area in zebrafish brain following SD (Additional file 2**:** Fig. S2A). Recovery following SD did not lead to the resolution of the increased expression of GFAP (Additional file 2: Fig. S2A). Instead, RSD induced a further elevation in the expression of GFAP and S100 calcium-binding protein β (S100β), another astrocyte activation marker, compared to SD(R) (Fig. 1C). SD induced activation of the nuclear factor-κB (NF-κB) pathway, as indicated by degradation of inhibitor of κBα (IκBα), which returned to baseline levels after the recovery period (Additional file 2: Fig. S2B). However, RSD induced persistent activation of NF-κB, as evidenced by not only IκBα degradation but also increased nuclear translocation of the p65 subunit of NF-κB, even after the recovery period, in the Dl region of zebrafish brain (Fig. 1D). Accumulation of pathological markers of Alzheimer’s disease (AD), such as Aβ and pTau, was observed following SD(R) or RSD. Specifically, RSD induced an increase in Aβ accumulation with no significant changes in pTau levels. On the other hand, no significant alterations in Aβ levels were observed in the brains of subjects in the SD(R) group (Fig. 1E).

### RSD leads to a decrease in brain glucose levels in adult zebrafish

**Fig. 2:**
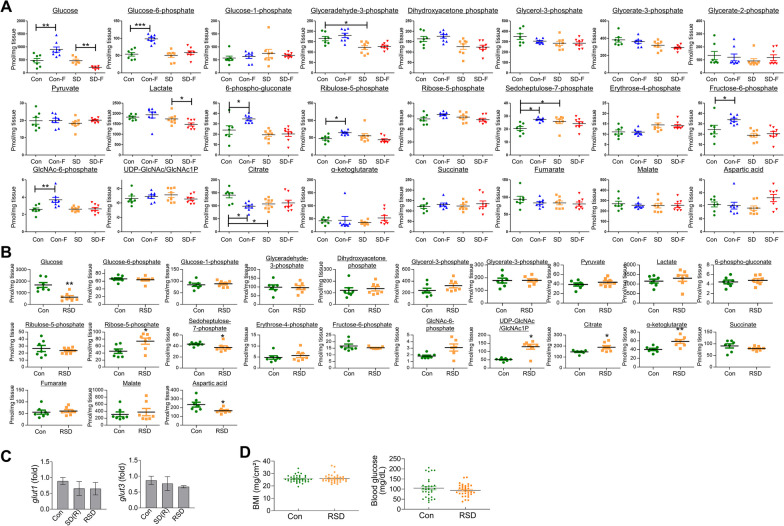
Changes in glucose metabolites in brains of control, SD and RSD zebrafish. **A** Graphs represent quantification of selected glucose metabolites by GC–MS/MS in the brains of control and SD zebrafish with or without the fear context. A fear context memory test was conducted after the SD. After a 6-h interval following the memory test, the brains were sacrificed for metabolic analysis. The Mann–Whitney *U* test was used for statistical comparisons of groups (*p < 0.05 and **p < 0.01). Cont, control; Con-F, 6 h after fear context in control group; SD, sleep-deprivation; SD-F, 6 h after fear context in SD group. **B** Graphs represent quantification of selected glucose metabolites using GC–MS/MS in the brains of control and RSD zebrafish. Statistical analysis was carried out by Mann–Whitney *U* test (*p < 0.05 and **p < 0.01 versus Con). **C** mRNA expression levels of pooled brain samples (*n* = 3/group) were measured using quantitative real-time PCR with specific primers. The data represent the mean ± SEM of target gene expression normalized to β-actin levels using the delta–delta Ct method. The experiments were independently replicated three times. Statistical analysis was performed using the Kruskal–Wallis test (*n.s*). **D** Zebrafish BMI and blood glucose levels were analyzed and compared with the control group following RSD induction. The height and weight of fish were measured to calculate BMI using the formula BMI = mg/cm^2^. Blood glucose levels were measured from the zebrafish tail blood samples. The values are displayed as the mean ± SEM, and individual values are represented by dots (*n* = 30 ~ 36/group). Statistical analysis was performed using the Mann–Whitney test (*n.s*)

Our previous findings established a potential link between SD-induced cognitive impairment and hexosamine biosynthetic pathway (HBP) dysregulation of glucose metabolism in the brain [27, 35]. The concentrations of major glucose metabolites in brains of control and sleep-deprived zebrafish with or without the fear context stimulation were analyzed via gas chromatography–mass spectrometry (GC–MS). The glucose levels were not significantly changed under conditions of SD. The brain glucose level was markedly increased after fear stimulation in the control group but decreased in response to the fear context in SD zebrafish. The pentose phosphate pathway (PPP) metabolite, sedoheptulose-7-P, was increased in the brains of SD zebrafish relative to control, while 6-P-gluconate, ribulose-5-P and sedoheptulose-7-P were increased in response to fear context stimulation in the control but not SD group. Moreover, SD did not induce significant changes in HBP metabolites. Interestingly, major HBP metabolites, including glucose-6-P, fructose-6-P, and GlcNAc-6-P, were significantly increased in response to fear stimulation in the brains of control but not sleep-deprived zebrafish (Fig. 2A). Notably, UDP–GlcNAc and GlcNAc-1-P could not be separated via GC–MS in this study and require the application of liquid chromatography (LC)–MS for separation in future studies.

Next, we investigated the changes in brain glucose metabolites induced by RSD. The brain glucose level was significantly decreased in zebrafish subjected to RSD. Among the TCA cycle metabolites examined, citrate and α-ketoglutarate levels were increased, while aspartic acid was decreased under RSD relative to the control group (Fig. 2B). Levels of UDP–GlcNAc/GlcNAc-1-P, HBP metabolites, were additionally increased in RSD compared to control brain. Examination of the mRNA levels of major glucose transporters of the blood–brain barrier [36], *glut1* and *glut3*, in brain tissue revealed no significant changes between the RSD and control groups (Fig. 2C). In addition, we observed no changes in body mass index (BMI) or levels of glucose in peripheral blood (Fig. 2D).

### RSD modulates O-GlcNAc cycling in zebrafish brain

**Fig. 3:**
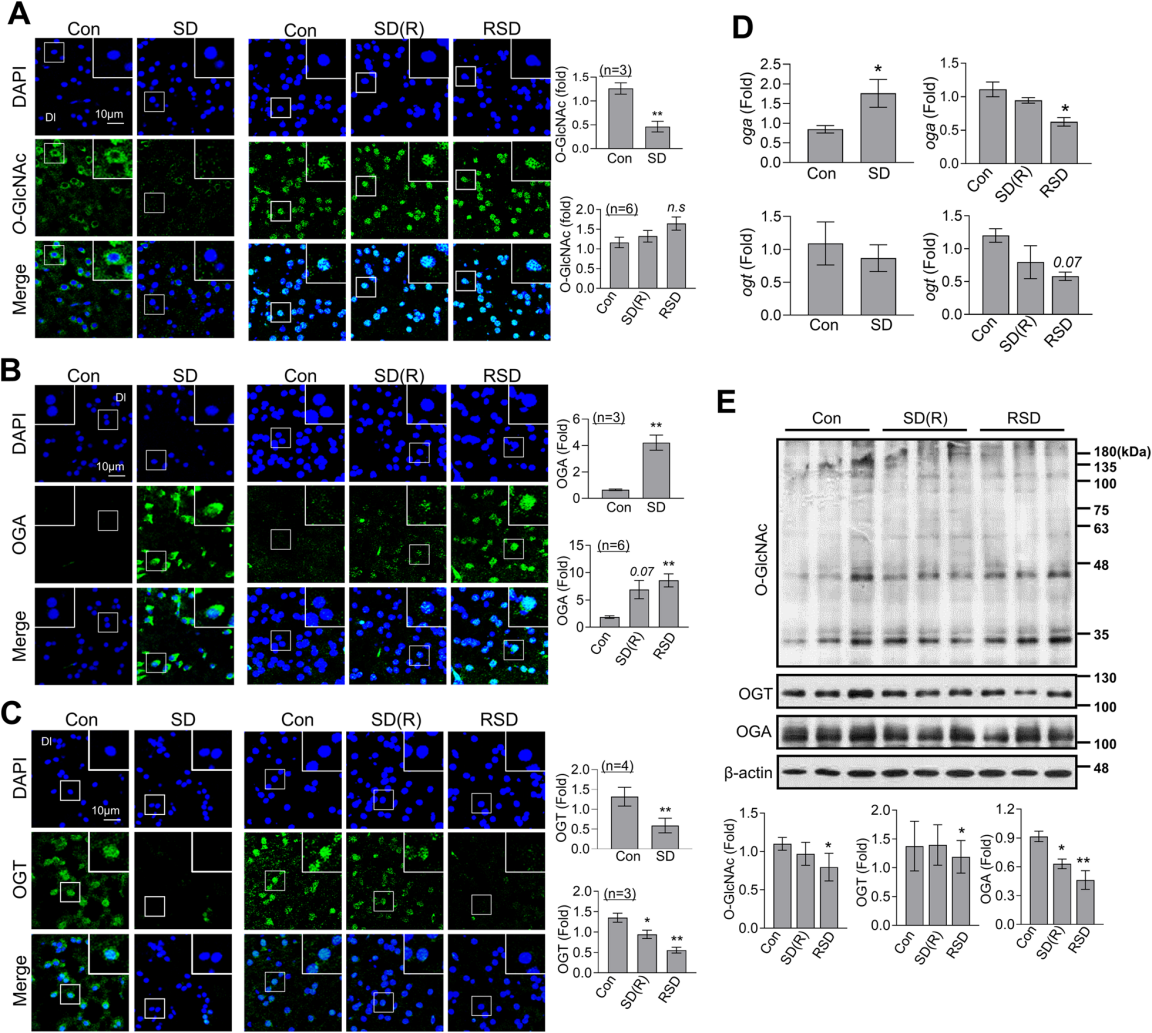
OGT, OGA and *O*-GlcNAcylation changes following SD or RSD. The zebrafish brains were sacrificed for analysis after the induction of SD, SD(R), or RSD. **A**–**C** Representative confocal images (× 40) of DAPI (blue), *O*-GlcNAc (**A**, green), OGA (**B**, green), OGT (**C**, green), and merged immunofluorescence staining are shown for the Dl region of the zebrafish telencephalon. Enlarged images are presented within white boxes. The graphs represent the quantitative results for each antibody, normalized to the DAPI levels (*n* = 3 ~ 6/group). **D** mRNA expression levels of pooled brain samples (*n* = 3/group) were measured using quantitative real-time PCR with specific primers. Data represent the mean ± SEM of target gene expression, normalized to β-actin levels using the delta–delta Ct method. The experiments were independently replicated three times. **E** Representative Western blot images of *O*-GlcNAc, OGT, OGA, and β-actin from zebrafish whole brains (*n* = 9/group). The graphs represent the quantitative results for each antibody, normalized to the β-actin levels. For statistical analysis, the Mann–Whitney test was performed for the two group comparisons, while the Kruskal–Wallis test with the original Benjamini and Hochberg false FDR was used for comparisons involving more than three groups (*p < 0.05, **p < 0.01 versus Con)

We further investigated the *O*-GlcNAcylation patterns and expression levels of OGT and OGA in zebrafish brain. SD induced a decrease in *O*-GlcNAcylation, along with downregulation of OGT and upregulation of OGA, in the Dl region of zebrafish brain (Fig. 3A–C). After 4 days of recovery following SD, *O*-GlcNAcylation was almost restored to control levels. However, the decrease in brain OGT level and increase OGA level after SD was not reversed after the restoration period in the SD(R) group. Unexpectedly, RSD did not induce significant changes in *O*-GlcNAcylation levels despite the observed decrease in OGT and increase in OGA in the Dl region of zebrafish brain (Fig. 3A–C). The *oga* mRNA level in brain was increased after SD but it suppressed under conditions of both RSD. Interestingly, however, while OGT protein expression was significantly decreased by both SD and RSD, the *ogt* mRNA level was not significantly altered by SD and only a slight decrease was evident in the RSD group (Fig. 3D). Western blot analysis in whole brain following RSD revealed a decrease in the levels of *O*-GlcNAc levels. In addition, RSD induced with a decrease in whole brain OGT protein expression (Fig. 3E). It is important to note that the observed decrease in total OGA protein level in the brain was inconsistent with the immunofluorescence finding of increased OGA levels following both SD(R) and RSD.

### GlcN relieves RSD-induced impairment of L/M, astrocyte activation, and Aβ accumulation

**Fig. 4:**
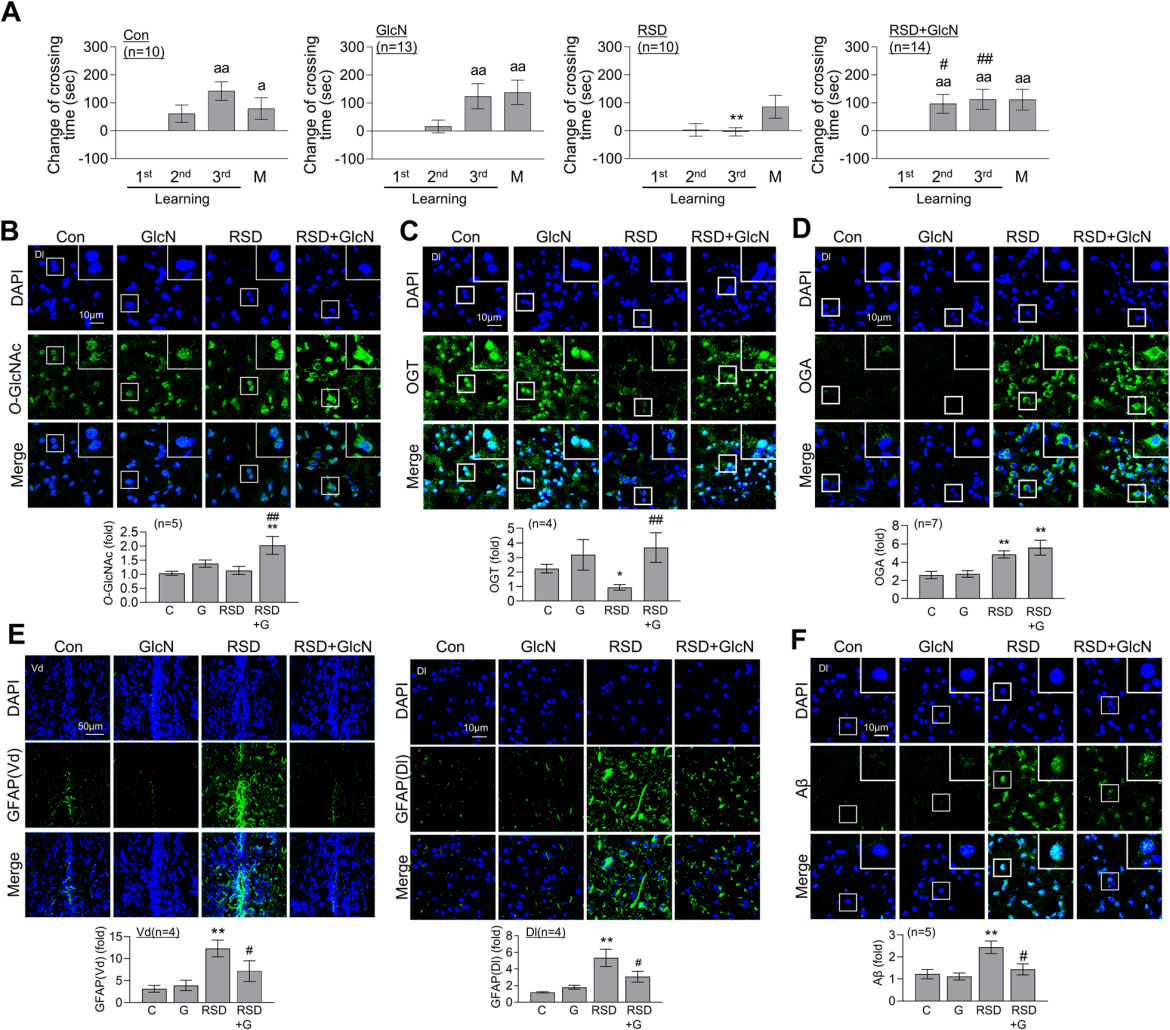
Restoration of RSD-induced L/M deficit, astrocyte activation, and Aβ accumulation by GlcN treatment. Zebrafish were intraperitoneally injected with GlcN (20 μg/g) on the day of SD exposure during each episode of SD throughout 5 cycles of RSD. After the final RSD induction, the fear context L/M test and brain analysis were conducted. **A** Graphs depict the results of fear context L/M tests conducted after the RSD induction, comparing groups with or without GlcN injection. The graphs represent the altered crossing times compared to those observed during the first learning session and display the mean ± SEM (*n* = 10 ~ 14/group). For within-group comparisons, statistical analysis was performed using the Friedman ANOVA test with the original Benjamini and Hochberg FDR (^a^*p* < 0.05, ^aa^*p* < 0.01 versus 1st learning). A between-group comparison was conducted using a two-way ANOVA with Tukey’s multiple comparisons test (**p < 0.01 versus Con, ^#^*p* < 0.05, ^##^*p* < 0.01 versus RSD). **B**–**F** Representative confocal images (× 40) of DAPI (blue), GFAP (**B**, green), *O*-GlcNAc (**C**, green), OGT (**D**, green), OGA (**E**, green), Aβ (**F**, green), and merged immunofluorescence staining are shown for the Dl and Vd regions of the zebrafish telencephalon. Enlarged images are presented within white boxes. The graphs display the quantitative results for each antibody, normalized to the DAPI levels (*n* = 4 ~ 7/group). For statistical analysis, the Kruskal–Wallis test with the original Benjamini and Hochberg FDR was performed (*p < 0.05, **p < 0.01 versus Con, ^#^*p* < 0.05, ^##^*p* < 0.01 versus RSD)

Next, we examined the functional and molecular changes in brain induced by RSD following treatment with GlcN, which acts as a direct substrate for *O*-GlcNAcylation, bypassing the HBP. GlcN (20 μg/g) was administered intraperitoneally 12 h before the start of each cycle of SD and significantly alleviated RSD-induced impairment of fear context L/M function (Fig. 4A). Interestingly, *O*-GlcNAc levels in the Dl region of zebrafish brain did not change in response to GlcN alone but were elevated by GlcN under RSD conditions compared to both control and RSD (Fig. 4B). GlcN administration reversed the RSD-induced decrease in OGT in brain but had no significant effect on RSD-induced increase in OGA (Fig. 4C, andD). GlcN inhibited the RSD-induced increase in GFAP levels in both Vd and Dl regions (Fig. 4E) and Aβ accumulation in the Dl region (Fig. 4F) of zebrafish brain. Notably, repeated injections (5 cycles with 3 days of GlcN and 4 days of restoration without GlcN in between) with a higher dose of GlcN (200 μg/g) induced defects in fear context L/M function in zebrafish (Additional file 3: Fig. S3).

### DON induces brain dysfunction and decreases O-GlcNAcylation in the brain

**Fig. 5:**
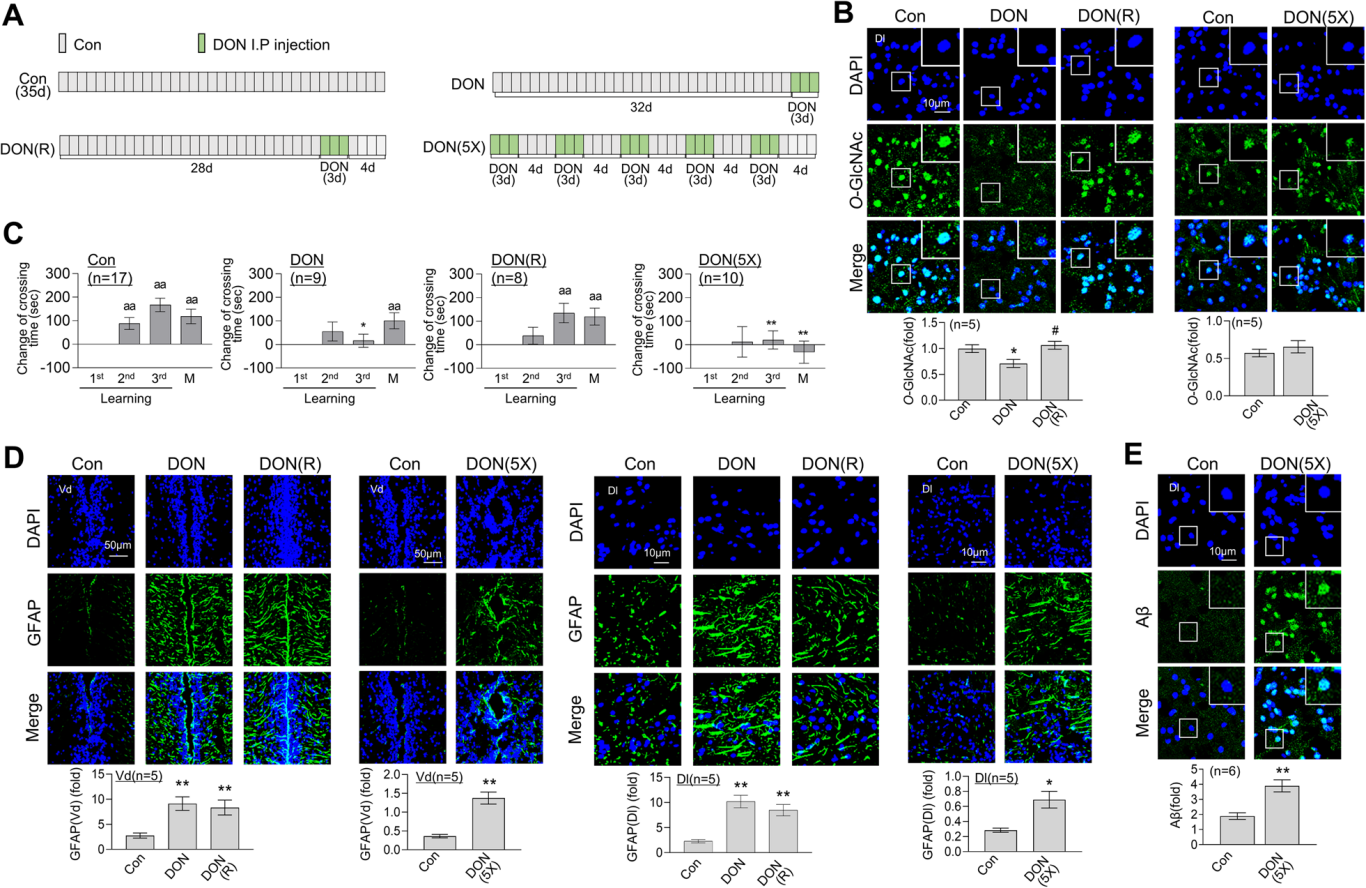
Induction of L/M dysfunction, *O*-GlcNAcylation decrease, and enhanced astrocyte activation and Aβ accumulation by DON treatment. DON (500 ng/g) was administered via single or multiple cycles of intraperitoneal injections: three consecutive DON treatments (DON), 4 days of recovery without DON following the initial treatment (DON (R)), and a total of 5 cycles consisting of 3 days of DON injections with 4 days of recovery in between (DON (5X)). A day after the final cycle of DON injections, the fear context L/M test or brain analysis was conducted. **A** Schematic experimental design and procedure of DON, DON (R), and DON (5X). **B** Representative confocal images (× 40) of DAPI (blue), *O*-GlcNAc (green), and merged are immunofluorescence staining of the Dl region of zebrafish telencephalon. Enlarged images are presented in the white boxes. The graphs represent the quantitative results for each antibody with normalization by the DAPI levels (*n* = 5 ~ 6/group). Statistical analysis was performed using the Kruskal–Wallis test with the original FDR of Benjamini and Hochberg (*p < 0.05 versus Con, ^#^*p* < 0.05 versus DON). **C** Graphs represent the results of fear context L/M test after different schemes of DON injection. They depict the altered crossing times compared to those observed during the first learning session and display the mean ± SEM (*n* = 8 ~ 17/group). For statistical analysis, a within-group comparison was performed using the Friedman ANOVA test with the original FDR of Benjamini and Hochberg (^a^*p* < 0.05, ^aa^*p* < 0.01 versus 1st learning). Between the group comparison, a two-way ANOVA with Tukey’s multiple comparisons test was performed (*p < 0.05, **p < 0.01 versus Con). (**D** and **E**) Representative confocal images (40x) of DAPI (blue), GFAP (**D**, green), Aβ (**E**, green), and merged immunofluorescence staining in the Dl and Vd regions of zebrafish telencephalon are shown. Enlarged images are presented in the white boxes. The graphs represent the quantitative results for each antibody with normalization by the DAPI levels (*n* = 5 ~ 6/group). For statistical analysis, the Mann–Whitney test was performed the two-group comparisons, while the Kruskal–Wallis test with the original Benjamini and Hochberg false FDR was used for comparisons involving more than three groups (*p < 0.05, **p < 0.01 versus Con)

To determine the link between dysregulation of HBP/*O*-GlcNAc cycling and neurodegeneration, we investigated the effect of pharmacological inhibition of HBP entry on brain function using the glutamine–fructose-6-phosphate amidotransferase (GFAT)-specific inhibitor, 6-diazo-5-oxo-L-norleucine (DON). DON was administered intraperitoneally for three consecutive days, followed by a 4-day recovery period (DON(R)). Alternatively, DON(R) treatment was repeated five times over a span of 5 weeks to establish a model of chronic DON exposure (DON(5X)) (Fig. 5A). A single episode of DON treatment led to a decrease in *O*-GlcNAc levels in the Dl region of zebrafish brain. However, after a 4-day recovery period, *O*-GlcNAc returned to control levels. Repeated treatment with DON induced no significant changes in brain *O*-GlcNAc levels relative to control (Fig. 5B). To assess the effects of DON on behavior and cognitive function, the fear context L/M, T-maze, and NTT tests were conducted. DON treatment led to significant impairment of L/M compared to the control, which was restored after 4 days (Fig. 5C). Furthermore, DON(5X) treatment resulted in severe L/M impairment (Fig. 5C). In the T-maze test, no significant differences in spatial memory and social preference were observed between the DON(5X) and control groups (Additional file 4: Fig. S4A). Analysis of NTT revealed that swimming behavior patterns and anxiety remained unchanged between the DON, DON(5X), and control groups (Additional file 4: Fig. S4B, C). Next, we examined whether DON induces astrocyte activation. Both single and repeated treatments of DON significantly promoted GFAP expression in the Vd and Dl regions of zebrafish brain (Fig. 5D). However, the restoration period did not lead to resolution of DON-induced GFAP activation. Interestingly, repeated DON treatment led to substantial accumulation of Aβ in zebrafish brain (Fig. 5E).

### GlcN reverses DON-induced brain dysfunction

**Fig. 6:**
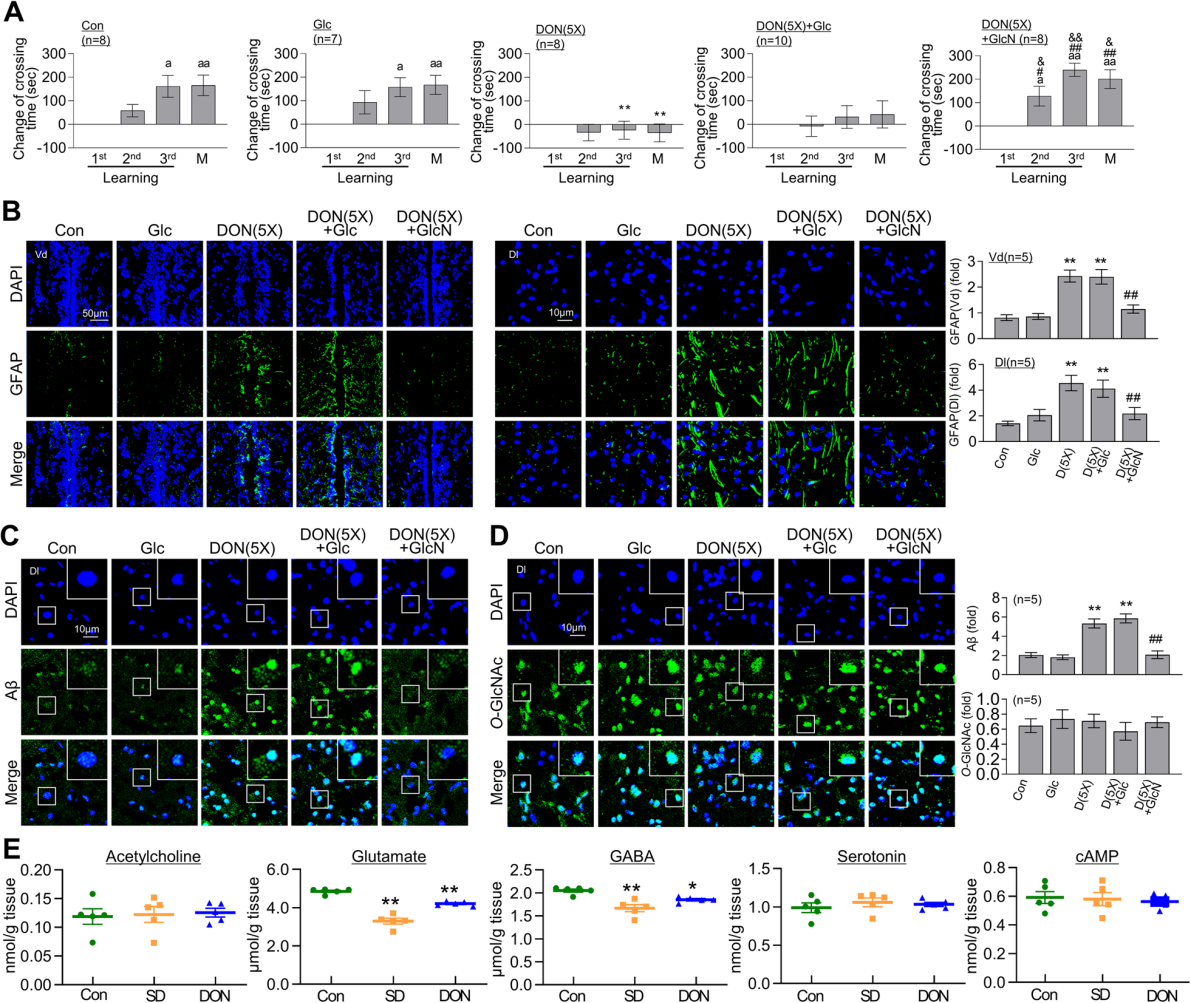
Restoration of DON induced L/M defect, astrocyte activation and Aβ accumulation by GlcN. DON (500 ng/g) was intraperitoneally injected for 5 cycles, with each cycle consisting of 3 days of injection followed by 4 days of recovery (DON (5X)). Some groups received an additional intraperitoneal injection of glucose (Glc, 20 μg/g) or GlcN (20 μg/g). After the final cycle of DON injections, fear context L/M test or brain analysis was conducted. **A** Graphs represent the results of the fear context L/M test for the DON (5X) group with or without Glc or GlcN injection. They illustrate the altered crossing times compared to those observed during the first learning session and display the mean ± SEM (*n* = 7 ~ 10/group). For within-group comparisons, the Friedman ANOVA test was performed with the original FDR correction method of Benjamini and Hochberg. (^a^*p* < 0.05, ^aa^*p* < 0.01 versus 1st learning). For between-group comparisons, a two-way ANOVA with Tukey’s multiple comparisons test was performed (*p < 0.05, **p < 0.01 versus Con, ^#^*p* < 0.05, ^##^*p* < 0.01 versus DON (5X), ^&^*p* < 0.05, ^&&^*p* < 0.01 versus RD + Glc). **B**–**D** Representative confocal images (40×) of DAPI (blue), GFAP (**B**, green), Aβ (**C**, green), *O*-GlcNAc (**D**, green), and merged immunofluorescence staining in the Dl and Vd regions of zebrafish telencephalon are displayed. Enlarged images are presented in the white boxes. The graphs represent the quantitative results for each antibody with normalization by the DAPI levels (*n* = 5/group). For statistical analysis, the Kruskal–Wallis test with the original FDR of Benjamini and Hochberg was performed (*p < 0.05, **p < 0.01 versus Con, ^##^*p* < 0.01 versus DON (5X)). **E** The graphs represent the levels of acetylcholine, glutamate, GABA, serotonin, and cAMP in zebrafish brains (*n* = 5/sample) 1 day after SD or DON treatment. For statistical analysis, the one-way ANOVA with post hoc Tukey’s multiple comparison tests were performed. The data are presented as the mean ± SEM (*p < 0.05, **p < 0.01 versus Con)

We investigated the effects of glucose and GlcN on DON-induced neuronal dysfunction. DON inhibits the entry of glucose into the HBP pathway, which can be bypassed by GlcN, facilitating direct entry into HBP [37]. Glucose or GlcN was administered intraperitoneally at each cycle of DON treatment. Glucose administration did not affect the dysfunction induced by repeated DON in fear context memory. However, GlcN effectively suppressed DON-induced impairment of L/M (Fig. 6A). Furthermore, the increase in GFAP expression induced by DON(5X) in the Vd and Dl regions of zebrafish brain was not affected by glucose but effectively suppressed by GlcN (Fig. 6B). Importantly, GlcN specifically inhibited accumulation of Aβ induced by DON(5X), while glucose had no effect on Aβ deposition (Fig. 6C). Neither glucose nor GlcN had a significant impact on brain *O*-GlcNAc levels, both with and without repeated DON treatment (Fig. 6D). Analysis and comparison of neurotransmitter levels in the SD- and DON-treated groups revealed a selective decrease in glutamate and GABA relative to the control group (Fig. 6E).

### OGT overexpression in zebrafish brain restores L/M dysfunction induced by RSD

**Fig. 7:**
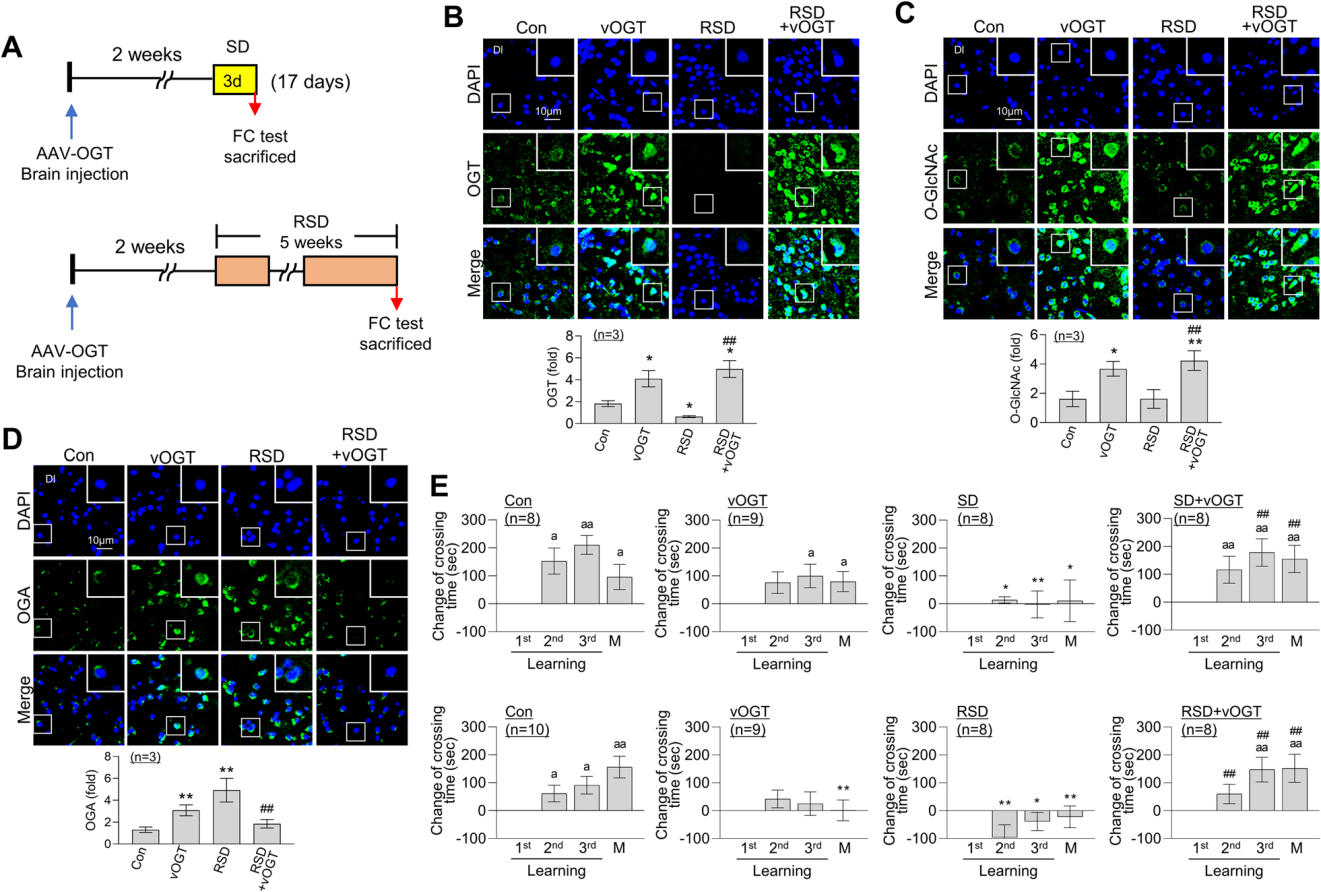
Restoration of RSD-induced L/M deficit by overexpression of OGT. An vOGT virus was injected into the brains of zebrafish. **A** Schematic illustrating the experimental design of vOGT (vOGT) injection and induction of either SD or RSD. **B**–**D** Representative confocal images (40×) of DAPI (blue), OGT (**B**, green), *O*-GlcNAc (**C**, green), OGA (**D**, green), and merged immunofluorescence staining in the Dl region of zebrafish telencephalon are displayed. Enlarged images are presented in the white boxes. The graphs represent the quantitative results for each antibody with normalization by the DAPI levels (*n* = 3/group). For statistical analysis, the Kruskal–Wallis test with the original FDR of Benjamini and Hochberg was performed (*p < 0.05, **p < 0.01 versus Con, ^##^*p* < 0.01 versus RSD). **E** Graphs represent fear context L/M test results for the SD or RSD group with or without vOGT injection. They illustrate the altered crossing times compared to those observed during the first learning session and display the mean ± SEM (*n* = 8 ~ 10/group). For statistical analysis, within-group comparisons, the Friedman ANOVA test was performed with the original FDR of Benjamini and Hochberg (^a^*p* < 0.05, ^aa^*p* < 0.01 versus 1st learning). Between-group comparisons, a two-way ANOVA with Tukey’s multiple comparisons test was performed (*p < 0.05, **p < 0.01 versus Con, ^##^*p* < 0.01 versus SD or RSD)

A recombinant adeno-associated virus serotype 8 (AAV8) cassette containing the murine OGT gene (vOGT) was injected into the inner part of zebrafish brain skull 2 weeks before SD or RSD, resulting in a total experimental duration of 17 days and 7 weeks, respectively (Fig. 7A). Administration of vOGT led to a significant increase in expression of OGT and *O*-GlcNAc in zebrafish brain, as confirmed based on immunofluorescence signals (Additional file 5: Fig. S5A, B, C). No changes in the OGA level were observed following this procedure (Additional file 5: Fig. S5D). The SD-induced decrease in OGT and *O*-GlcNAc was reversed following administration of vOGT (Additional file 5: Fig. S5E, F). Moreover, the increase in OGA following SD was reduced by vOGT (Additional file 5: Fig. S5G). As shown in Fig. 7B, expression of OGT in zebrafish brain remained elevated after 7 weeks of vOGT injection compared to the control group. In addition, vOGT effectively reversed the decrease in OGT caused by RSD in the Dl region of zebrafish brain. Consistent with this finding, we observed a marked increase in *O*-GlcNAcylation induced by vOGT in both the control and RSD groups (Fig. 7C). Unexpectedly, administration of vOGT resulted in an increase in OGA expression after 7 weeks (Fig. 7D). However, under conditions of RSD, vOGT effectively counteracted the RSD-induced increase in OGA levels within the brain (Fig. 7D). Notably, while vOGT alone significantly impaired L/M function 7 weeks after the injection compared to control group, L/M dysfunction induced by either SD or RSD was successfully reversed (Fig. 7E).

### OGT overexpression in zebrafish brain suppresses GFAP increase and Aβ accumulation induced by RSD

**Fig. 8:**
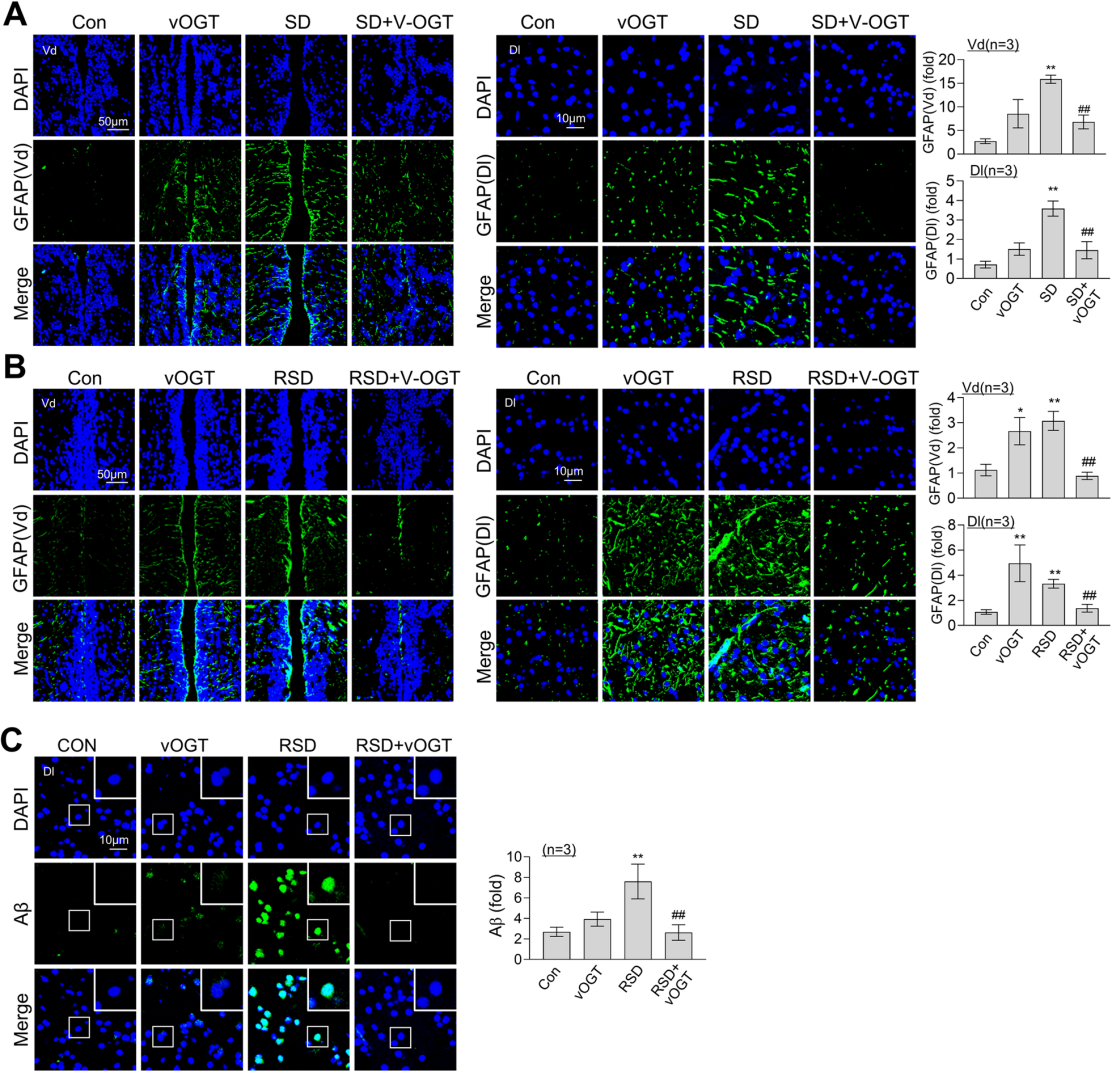
Suppression of RSD-induced astrocyte activation and Aβ accumulation by overexpression of OGT. An vOGT virus was injected into the brains of zebrafish. Representative confocal images (40×) of DAPI (blue), GFAP (**A** and **B**, green), Aβ (**C**, green), and merged immunofluorescence staining in the Dl or Vd regions of zebrafish telencephalon are displayed. Enlarged images are presented in the white boxes. The graphs represent the quantitative results for each antibody with normalization by the DAPI levels (*n* = 3/group). For statistical analysis, the Kruskal–Wallis test with the original FDR of Benjamini and Hochberg was performed (*p < 0.05,  **p < 0.01 versus Con, ^##^*p* < 0.01 versus SD or RSD)

We conducted additional investigations to explore the impact of OGT overexpression on GFAP increase and Aβ accumulation induced by RSD. Zebrafish brains were injected with the vOGT virus, resulting in increased GFAP expression after 7 weeks. However, intriguingly, OGT overexpression suppressed the GFAP increase caused by SD or RSD in both the ventral and dorsal regions of the zebrafish brain (Fig. 8A, andB). Moreover, administration of vOGT significantly suppressed RSD-induced Aβ accumulation in the Dl region of zebrafish brain (Fig. 8C).

### SD decreases O-GlcNAcylation of amyloid precursor protein (APP) and increases the expression and activity of β-secretase

**Fig. 9:**
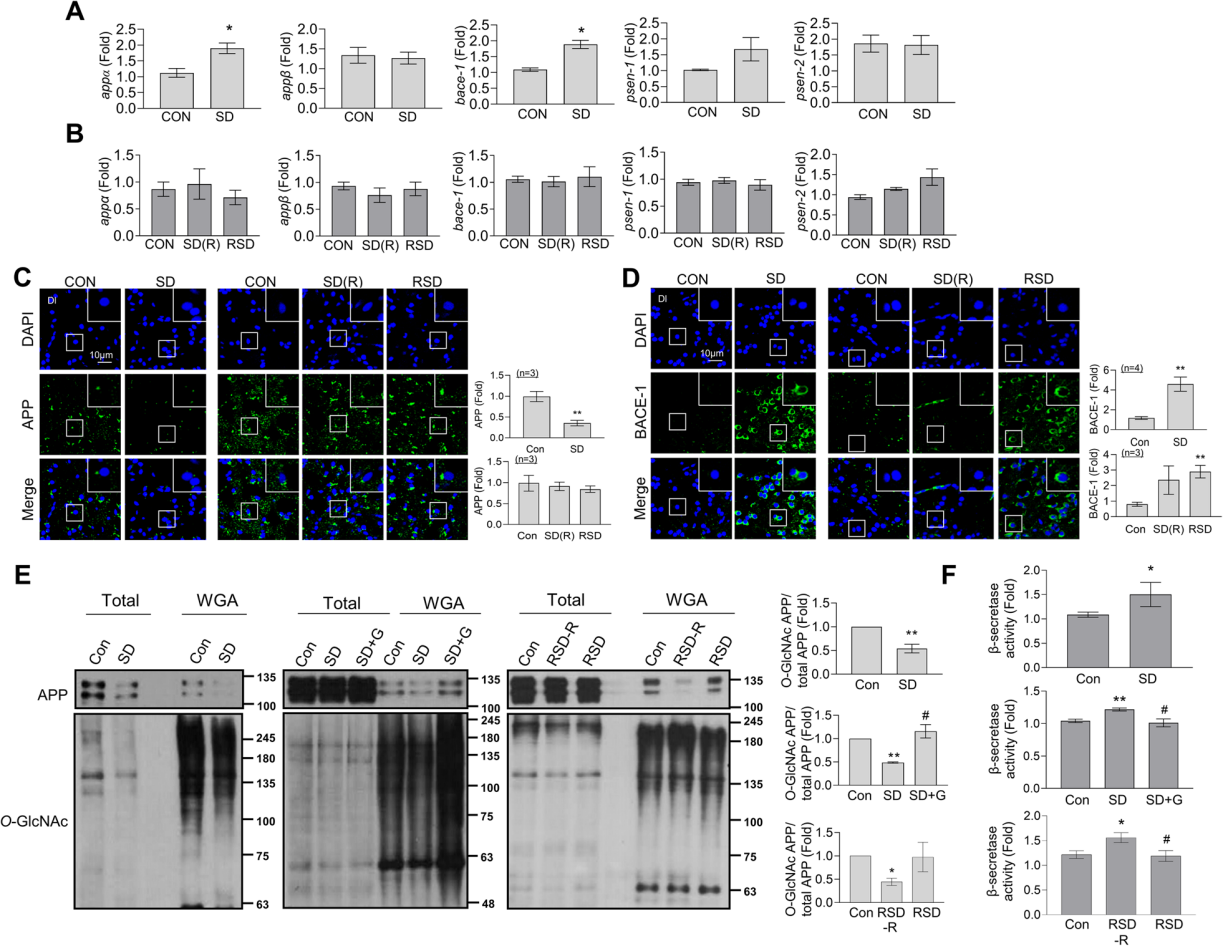
SD-induced increase in mRNA levels of APP and β-secretase expression, and decrease in APP *O*-GlcNAcylation. Zebrafish brains were analyzed after exposure to SD or RSD, with or without a recovery period, as well as after RSD without recovery (RSD-R). In some conditions, intraperitoneal GlcN (20 μg/g) was administered. After the induction of SD, SD(R), RSD-R, or RSD, the zebrafish brains were sacrificed. **A, B** mRNA expression levels of pooled brain samples (*n* = 3/group) were measured using quantitative real-time PCR with specific primers. Values are presented as the mean ± SEM for each target gene expression, normalized to β-actin levels using the delta–delta Ct method. The experiments were independently replicated three times. For statistical analysis, the Mann–Whitney test was used for the two-group comparisons, and the Kruskal–Wallis test with the original FDR of Benjamini and Hochberg was used for comparisons involving more than three groups (*p < 0.05 versus Con). **C** Representative confocal images (40x) of DAPI (blue), APP (**C**, green), BACE-1 (**D**, green), and merged immunofluorescence staining in the Dl region of zebrafish brain are displayed. Enlarged images are presented in the white boxes. The graphs represent the quantitative results for each antibody with normalization to the DAPI levels (*n* = 3 ~ 4/group). For statistical analysis, the Mann–Whitney test was performed the two-group comparisons, while the Kruskal–Wallis test with the original Benjamini and Hochberg false FDR was used for comparisons involving more than three groups (**p < 0.01 versus Con). **D** WGA pull-down assays were performed using total protein extracted from whole brains of zebrafish (3 brain pools per group). The total cell lysates were incubated with WGA agarose, and the proteins bound to WGA were analyzed using Western blot assay with specific antibodies. The graphs represent the quantitative results for the level of WGA-bound APP, normalized to the total APP levels. The experiments were independently replicated three times. For statistical analysis, the Mann–Whitney test was performed (*p < 0.05, **p < 0.01 versus Con, ^#^*p* < 0.05 versus SD). **E** β-Secretase activities were measured in the whole brain of zebrafish (*n* = 4 ~ 5/group). Values are presented as the mean ± SEM. For statistical analysis, the Mann–Whitney test was performed for the two-group comparisons, and the Kruskal–Wallis test with the original FDR of Benjamini and Hochberg was performed for comparisons involving more than three groups (*p < 0.05, **p < 0.01 versus Con, #*p* < 0.05 versus SD or RSD-R)

Next, we examined the levels of amyloid precursor protein (APP) isoforms and APP cleavage enzymes. Transcript levels of *appα* and *bace-1* in zebrafish brain were specifically increased by SD (Fig. 9A), which returned to control levels after the restoration period. RSD induced no significant changes in either *appα* or *bace-1* (Fig. 9B). Furthermore, mRNA levels of *appβ*, *psne-1*, and *psne-2* were not affected by SD or RSD. Interestingly, in contrast to mRNA levels, protein expression of APP was decreased under SD. During the restoration period, APP protein expression recovered to control levels. Following RSD, APP was maintained at a similar level as the control group (Fig. 9C). Consistent with mRNA results, BACE-1 protein expression was increased by SD. After the sleep recovery period, immunofluorescence of BACE-1 was not significantly different from that of the control group (Fig. 9D). In contrast, RSD induced a significant increase in BACE-1 expression in the Dl region of zebrafish brain.

APP undergoes *O*-GlcNAcylation, which promotes its non-toxic processing [38]. Consistent with the decrease in overall nucleocytoplasmic *O*-GlcNAc levels induced by SD, we observed reduced *O*-GlcNAcylation of APP. The SD-induced decrease in APP *O*-GlcNAcylation was restored upon GlcN treatment (Fig. 9E). RSD induced no significant changes in APP *O*-GlcNAcylation. However, exposure to RSD without a final restoration period (RSD-R) led to a decrease in APP *O*-GlcNAcylation. Further examination of β-secretase activity supported these mRNA and protein expression patterns, specifically, increase by SD and mitigation by GlcN treatment or restoration (Fig. 9F).

## Discussion

The current study employed a 5-week RSD protocol. This RSD paradigm aimed to model sleep loss over time, as experienced by individuals suffering from intermittent and repeated sleep disruptions. The choice of a 5-week duration was based on its effectiveness in inducing pathological effects related to AD in zebrafish brain while avoiding subjecting the animals to unnecessarily prolonged aversive conditions. By employing this approach, we could obtain valuable insights into the long-term pathophysiology of RSD and the potential mechanisms underlying associated adverse effects. Our experiments showed that RSD induces persistent cognitive dysfunction, distinct from the apparent near-complete recovery observed after a single episode of SD. Furthermore, RSD markedly increased Aβ accumulation in zebrafish brain, manifesting an AD-like phenotype.

We analyzed changes in brain metabolites in response to fear context stimuli, with the aim of elucidating variations in glucose metabolites associated with learning processes. Although some changes in the metabolites of PPP were observed in response to SD or fear stimulation, our primary focus was investigation of metabolic alterations specifically related to HBP. In our previous publications, we presented evidence of increased *O*-GlcNAc cycling in the brains of both mice and zebrafish following the fear conditioning [27, 35]. In this study, fear conditioning specifically led to an increase in major metabolites of HBP, such as glucose, glucose-6-phosphate, fructose-6-phosphate, and GlcNAc-6-phosphate, in brains of control but not SD zebrafish. Although further research is necessary, our findings offer novel insights into the link between cognitive impairment and HBP flux in the SD group. Another intriguing finding is that while a single episode of SD did not exert a significant effect, RSD induced a marked reduction in brain glucose levels. In view of the observation that changes in peripheral blood glucose levels and BMI were not significant, it is likely that RSD modulates glucose entry into the brain. Indeed, previous studies have shown a decrease in glucose uptake in the brains of mice subjected to chronic sleep restriction, accompanied by a reduction in the expression of GLUT1 at the blood–brain barrier (BBB) [39]. However, our experiments showed that mRNA levels of major glucose transporters at the BBB, *glut1* and *glut3*, were not significantly changed by RSD in brain of zebrafish. Considering that glucose transport to the brain involves multiple interactions among solutes, transporters, enzymes, and signaling processes within the neurovascular unit at the BBB, the RSD-associated mechanisms affecting brain glucose levels require further investigation. We speculated that the decreased brain glucose levels after RSD could induce a decrease in HBP flux. Unexpectedly, major HBP metabolites were not significantly affected by RSD, while UDP–GlcNAc/GlcNAc-1P was increased. This result may be attributable to the dynamics of enzymes that regulate the final step of the HBP, OGT and OGA. The gene and protein expression patterns of OGT and OGA, regulated by RSD, are complex. Data on the mRNA and protein expression levels of OGT and OGA were inconsistent, and for OGA, discrepancies between western blot and immunohistochemistry results were observed. The differential expression patterns of proteins or mRNAs regulated by RSD in zebrafish brain may be attributed to the fact that immunohistochemical experiments assessed OGT or OGA specifically expressed in the Dl region, the hippocampus area of the zebrafish, while western blot and mRNA measurements were conducted using whole brain of zebrafish. Furthermore, unlike mRNAs, proteins exhibit differential expression patterns at specific timepoints due to various factors, such as post-transcriptional processes, half-life, and stability. Given that brain *O*-GlcNAcylation induced by SD or RSD returned to normal levels after a recovery period, we speculate that the *O*-GlcNAc cycling driven by the interplay between OGT and OGA during SD and restoration cycles is dynamic and reversible in nature. The accumulation of cyclic changes in *O*-GlcNAc during the repetition of SD may exert a cumulative effect on brain pathogenesis over time. At specific timepoints we observed, even though changes in *O*-GlcNAc levels may not be immediately evident, the cumulative effects of repetitive *O*-GlcNAc fluctuations occurring during the SD cycles could lead to a potential deterioration in brain function.

Our findings demonstrate that GlcN restores reduced *O*-GlcNAcylation, mitigates impaired L/M function and brain inflammation, and greatly reduces accumulation of Aβ. Using DON, we further confirmed the importance of HBP in maintaining normal brain function. Long-term and repetitive administration of DON induced repeated oscillation of *O*-GlcNAcylation in the brain, concurrently resulting in impaired L/M, brain inflammation, and accumulation of Aβ, similar to the symptoms of RSD. One common feature of repetitive SD- and DON-induced brain impairment is the presence of neuroinflammation and simultaneous restoration of cognitive function and suppression of neuroinflammation can be achieved through increased HBP flux. The issue of whether the HBP/*O*-GlcNAc pathway directly regulates neuroinflammation to induce cognitive dysfunction or if a common target regulates both neuroinflammation and cognitive function remains unclear. Although cognitive function impaired by SD recovered during the restorative phase, the sustained microglial and astrocyte activation suggests that acute brain inflammation alone does not directly mediate cognitive dysfunction. However, repetition of SD may lead to accumulation of neuroinflammatory responses, thus exacerbating cognitive dysfunction and deposition of Aβ. Another common feature between RSD and repeated DON treatment is the marked reduction in the neurotransmitters, GABA and glutamate, in both groups, compared to the control group. Previous reports have shown that concentrations of glutamate and GABA are significantly lower during sleep relative to wakefulness [40]. The physiological significance and mechanisms underlying the decrease in GABA and glutamate levels in brains of sleep-deprived zebrafish are not fully understood within the scope of the current study. Further investigation into the relationship between the expression and secretion of these neurotransmitters and HBP should provide valuable insights into the mechanisms underlying sleep-associated brain pathogenesis.

The overexpression of OGT in zebrafish brain confirmed a pivotal role of OGT in protecting against cognitive dysfunction induced by RSD through the maintenance of brain *O*-GlcNAc levels. Interestingly, the long-term overexpression of OGT paradoxically led to an increase in OGA, an enzyme with opposing actions. Similarly, it has been reported that OGA protein levels are downregulated in response to OGT inhibition, OGT knockdown, or OGT knockout in cultured cell lines [41–43]. Our findings suggest, for the first time, that the expression of OGT and OGA is tightly and dynamically regulated to maintain *O*-GlcNAc homeostasis in an in vivo system. While an ongoing debate exists regarding whether the increase in *O*-GlcNAcylation is beneficial or detrimental, we underscore the importance of maintaining homeostatic *O*-GlcNAc cycling in normal brain function. Our research showed that administration of GlcN at a concentration approximately ten times higher than the amount needed for restoring brain function after RSD resulted in the death of some zebrafish. Moreover, the surviving zebrafish exhibited significant impairment of fear context L/M function. Similarly, OGT overexpression induced impairment of L/M function despite its activity in restoring L/M function following damage by RSD. These findings highlight the essential regulatory role of *O*-GlcNAc homeostasis in normal brain function. Elucidation of the specific mechanisms by which this homeostatic maintenance regulates normal brain function will be a central focus of future studies.

Clarification of the potential role of HBP in accumulation of Aβ induced by RSD should provide a clearer understanding of the relationship between RSD and AD. The increase in mRNA levels of *app* and *bace-1* (encoding β-secretase) induced by SD may be highly relevant in the accumulation of Aβ during RSD. BACE-1 is responsible for cleaving APP and generating toxic forms of Aβ that are either 40 or 42 amino acids in length [44]. The levels of APP and β-secretase during RSD appear to be cyclically regulated, as they returned to normal during the subsequent normal sleep cycle. This cyclic fluctuation could potentially promote the processing and accumulation of toxic Aβ in the brain. APP is modified with *O*-GlcNAc [45] and increasing the level of *O*-GlcNAcylation level leads to elevated expression of soluble APPα and decreased toxic Aβ [46]. In the current study, we demonstrated that SD induces a reversible decrease in *O*-GlcNAcylation of APP, which can be restored through GlcN treatment. These findings strongly suggest a significant association between the *O*-GlcNAc levels of APP and its processing into toxic Aβ. However, direct investigation into the impact of mutations in the *O*-GlcNAc site of APP on SD-induced Aβ accumulation is necessary to definitively confirm this hypothesis.

In summary, the data provide novel evidence that RSD-associated L/M dysfunction and AD development is closely linked to the disruption of brain glucose metabolism via the HBP/*O*-GlcNAc cycling pathway. Our findings highlight cyclic changes in the expression of OGT and OGA, along with corresponding changes in *O*-GlcNAcylation levels, during the cycles of SD and recovery. In particular, RSD induced brain damage patterns similar to those observed with repeated blockage of the HBP pathway. Notably, increase in HBP flux induced through GlcN restored both functional and molecular changes in these two models. The dynamic and cyclic regulatory mechanisms of HBP flux, OGT, and OGA in accordance with sleep cycles remain important areas of focus for further research. Future studies should additionally aim to address the role of brain inflammation in disruption of HBP flux and associated mechanisms. Furthermore, identification of the specific *O*-GlcNAc sites that regulate accumulation of Aβ may be of importance in uncovering the pathways involved in AD progression. These collective investigations should provide valuable insights into the correlations among HBP dysregulation, brain inflammation, and pathogenesis of sleep-associated brain disorders, including AD.

### Supplementary Information

**Additional file 1: Figure S1. **Analyzing sleep–wake patterns, social ability, and anxiety in zebrafish induced by SD or RSD. The sleep–wake patterns, social abilities, and anxiety levels of zebrafish were evaluated after inducing either SD or RSD. (A) Sleep–wake patterns were assessed via the Actogram test. The representative single-plot bar graph illustrates zebrafish activity counts per hour (left). The average daily activity of the fish was quantified as percentages and is presented in the graph with mean ± SEM (*n* = 3 ~ 6) (right). Statistical analysis involved a one-way ANOVA followed by the Friedman test (*p < 0.05). (B) Social behavior was evaluated using the T-maze test. The graphs depict the running time (time taken to reach the friends’ zone), interaction time (duration of interaction near the friends’ zone), and latency (number of trials required to reach the correct arm). Statistical analysis was performed using two-way ANOVA with Tukey’s multiple comparisons test (*n* = 9 ~ 11/group, *n.s*). (C and D) Anxiety was measured using the NTT test in ASD or RSD zebrafish. (C) The heatmap images depict the distribution of zebrafish during novel tank exploration. The graphs illustrate the preference of zebrafish for the top (t), middle (m), and bottom (b) regions of the novel tank, displaying the mean ± SEM (*n* = 7 ~ 13/group). Two-way ANOVA followed by Tukey’s multiple comparisons test was performed for statistical analysis (*n.s*). (D) Representative graphs depict the average speed, mobility rate, total swim distance, number of freezing events, and average or total freezing time. The data represent the mean ± SEM (*n* = 7 ~ 13/group). Statistical analysis was conducted using two-way ANOVA followed by Tukey’s multiple comparisons test (*n.s*).

**Additional file 2: Figure S2. **SD-induced neuroinflammation in the brain of adult zebrafish. Zebrafish were exposed to extended light for 72 h (3 days). Representative confocal images (× 40) of DAPI (blue), GFAP (A, green), IκBα (B, green), and merged immunofluorescence staining are shown for the Vd or Dl region of the zebrafish brain. Enlarged images are presented within white boxes. The graphs display the quantitative results for each antibody with normalization to DAPI levels (*n* = 4 ~ 5/group). For statistical analysis, the Kruskal–Wallis test with the original Benjamini and Hochberg FDR was conducted (*p < 0.05, **p < 0.01 versus Con).

**Additional file 3: Figure S3. **Repeated GlcN injection with high-dose induces L/M deficit. Zebrafish were intraperitoneally injected with GlcN (200 μg/g) for 3 days and recovered 4 days and this cycle was repeated 5 times. The graphs represent fear context L/M test results. They illustrate the altered crossing times compared to those observed during the first learning session and display the mean ± SEM (*n* = 5 ~ 6/group). For within-group comparisons, the Friedman ANOVA test with the original FDR correction method by Benjamini and Hochberg was performed for statistical analysis (^a^*p* < 0.05, ^aa^*p* < 0.01 versus 1^st^ learning). For between-group comparisons, a two-way ANOVA with Tukey’s multiple comparisons test was performed for statistical analysis (*p < 0.05, **p < 0.01 versus Con).

**Additional file 4: Figure S4. **No changes in social ability and anxiety of the zebrafish after single or repeated DON treatment. Social ability and anxiety in adult zebrafish were assessed following single or repeated cycles of DON injections. DON (500 ng/g) was administered intraperitoneally for 3 consecutive days (DON). For the repeated episodes of DON injection, a cycle of 3 days of injection was followed by 4 days of recovery, and this cycle was repeated 5 times. A day after the final cycle of DON injections, behavior tests were conducted (DON (5X)). (A) Social behavior was assessed using the T-maze test. The graphs display the running time (time taken to reach the friends’ zone), interaction time (duration of interaction near the friends’ zone), and latency (number of trials required to reach the correct arm). Statistical analysis was conducted using a two-way ANOVA with Tukey’s multiple comparisons test (*n* = 10/group, *n.s*). (B and C) Anxiety was measured using the NTT test in DON or DON (5X) zebrafish. (B) The heatmap images represent the distribution during novel tank exploration. The graphs represent the tendency of zebrafish exploration in the top (t), middle (m), and bottom (b) of the novel tank with mean ± SEM (*n* = 10 ~ 15/group). For statistics analysis two-way ANOVA followed by Tukey multiple comparisons test was performed (*n.s*). (C) Representative graphs demonstrate the average speed, mobility rate, total swim distance, number of freezing events, and average or total freezing time. The data represent the mean ± SEM (*n* = 10 ~ 15/group). A two-way analysis of variance (ANOVA) was performed followed by Tukey’s multiple comparisons test for statistical analysis (*n.s*).

**Additional file 5: Figure S5. **Changes in OGT, OGA, and *O*-GlcNAcylation induced by OGT overexpression in the zebrafish brain. AAV8–CMV–mOGT–Myc–Flag (vOGT), a virus containing murine OGT overexpression construct, was injected into the zebrafish brain. Two weeks after the injection, zebrafish brains were sacrificed and analyzed. (A–D) Representative confocal images (40x) of DAPI (blue), Flag (A, green), OGT (B, green), *O*-GlcNAc (C, green), OGA (D, green), and merged images depict the immunofluorescence staining of the Dl regions of zebrafish telencephalon. Enlarged images are shown in the white boxes. The graphs present the quantitative results for each antibody, with normalization based on the DAPI levels (*n* = 3/group). For statistical analysis, the Kruskal–Wallis test with the original FDR correction method of Benjamini and Hochberg was performed (**p < 0.01 versus Con). (E–G) Two weeks after the vOGT injection, zebrafish were subjected to SD. Zebrafish brains were then sacrificed and analyzed after the SD. Representative confocal images (× 40) of DAPI (blue), OGT (E, green), *O*-GlcNAc (F, green), OGA (G, green), and merged are shown. These images depict the immunofluorescence staining of the Dl regions of zebrafish telencephalon. Enlarged images are presented in the white boxes. The graphs represent the quantitative results for each antibody, with the data normalized by the DAPI levels (*n* = 3/group). For statistical analysis, the Kruskal–Wallis test with the original FDR correction method by Benjamini and Hochberg was performed (**p < 0.01 versus SD).

## Data Availability

The data and materials used in this research are available upon request from the corresponding author.
