# Supplementary figures and images for: Repeated sleep deprivation decreases the flux into hexosamine biosynthetic pathway/O-GlcNAc cycling and aggravates Alzheimer’s disease neuropathology in adult zebrafish
Source: J Neuroinflammation. 2023 Nov 9;20:257. doi: 10.1186/s12974-023-02944-1 (PMC10634120; doi:10.1186/s12974-023-02944-1)

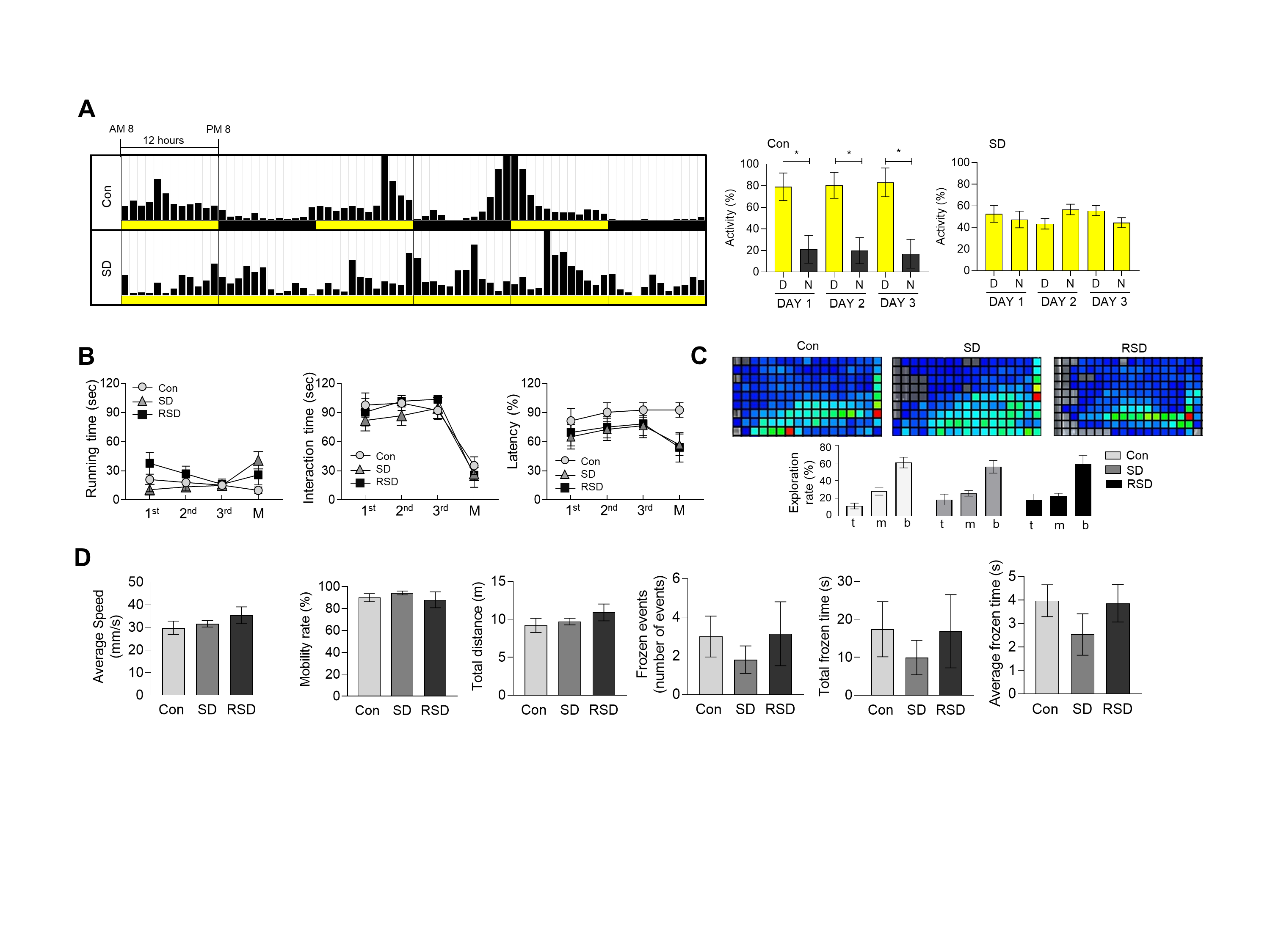

Supplement: Supplementary file 1 — Additional file 1: Figure S1. Analyzing sleep–wake patterns, social ability, and anxiety in zebrafish induced by SD or RSD. The sleep–wake patterns, social abilities, and anxiety levels of zebrafish were evaluated after inducing either SD or RSD. (A) Sleep–wake patterns were assessed via the Actogram test. The representative single-plot bar graph illustrates zebrafish activity counts per hour (left). The average daily activity of the fish was quantified as percentages and is presented in the graph with mean ± SEM (n = 3 ~ 6) (right). Statistical analysis involved a one-way ANOVA followed by the Friedman test (*p < 0.05). (B) Social behavior was evaluated using the T-maze test. The graphs depict the running time (time taken to reach the friends’ zone), interaction time (duration of interaction near the friends’ zone), and latency (number of trials required to reach the correct arm). Statistical analysis was performed using two-way ANOVA with Tukey’s multiple comparisons test (n = 9 ~ 11/group, n.s). (C and D) Anxiety was measured using the NTT test in ASD or RSD zebrafish. (C) The heatmap images depict the distribution of zebrafish during novel tank exploration. The graphs illustrate the preference of zebrafish for the top (t), middle (m), and bottom (b) regions of the novel tank, displaying the mean ± SEM (n = 7 ~ 13/group). Two-way ANOVA followed by Tukey’s multiple comparisons test was performed for statistical analysis (n.s). (D) Representative graphs depict the average speed, mobility rate, total swim distance, number of freezing events, and average or total freezing time. The data represent the mean ± SEM (n = 7 ~ 13/group). Statistical analysis was conducted using two-way ANOVA followed by Tukey’s multiple comparisons test (n.s). [file 12974_2023_2944_MOESM1_ESM.tif]

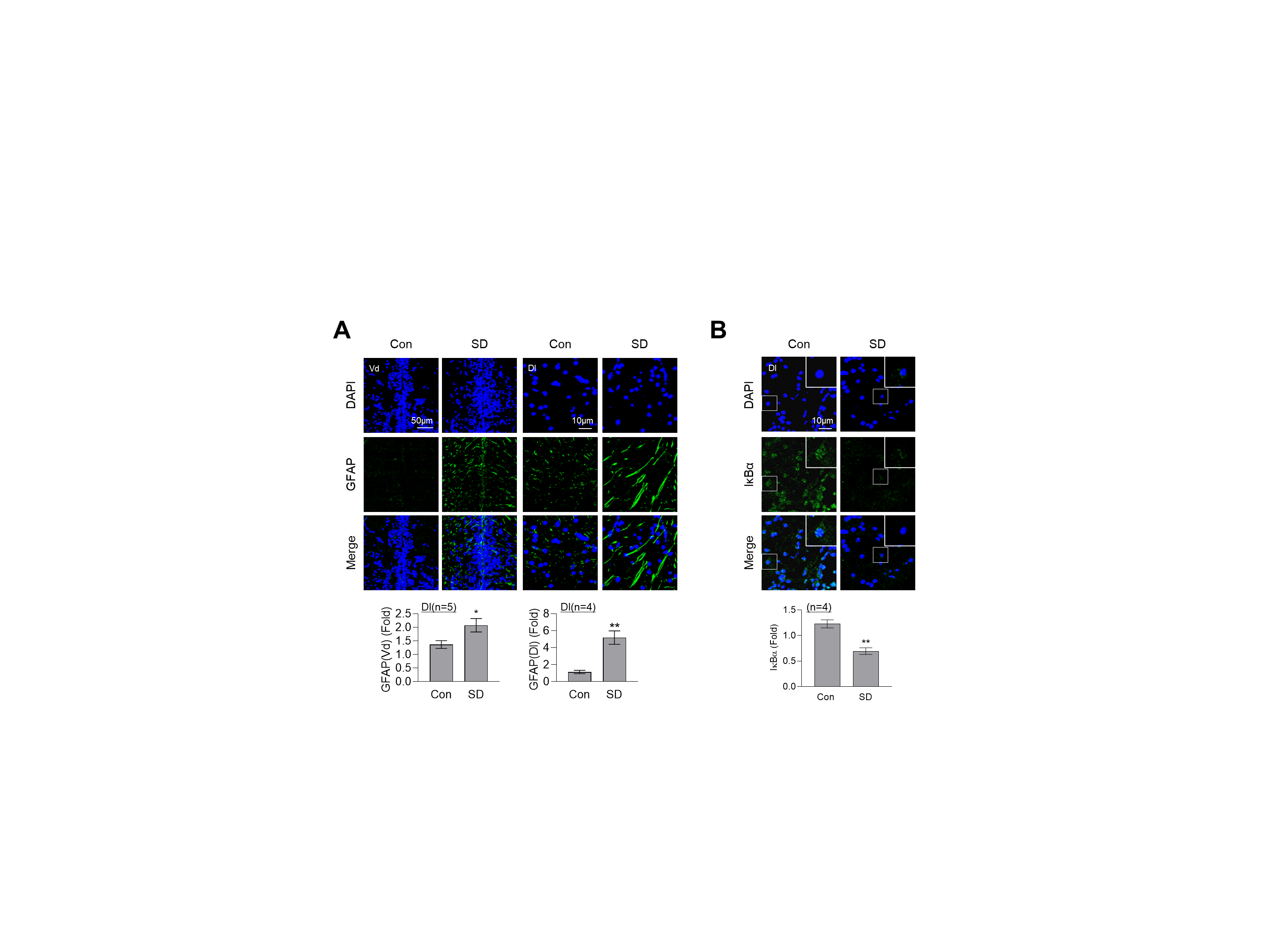

Supplement: Supplementary file 2 — Additional file 2: Figure S2. SD-induced neuroinflammation in the brain of adult zebrafish. Zebrafish were exposed to extended light for 72 h (3 days). Representative confocal images (× 40) of DAPI (blue), GFAP (A, green), IκBα (B, green), and merged immunofluorescence staining are shown for the Vd or Dl region of the zebrafish brain. Enlarged images are presented within white boxes. The graphs display the quantitative results for each antibody with normalization to DAPI levels (n = 4 ~ 5/group). For statistical analysis, the Kruskal–Wallis test with the original Benjamini and Hochberg FDR was conducted (*p < 0.05, **p < 0.01 versus Con). [file 12974_2023_2944_MOESM2_ESM.tif]

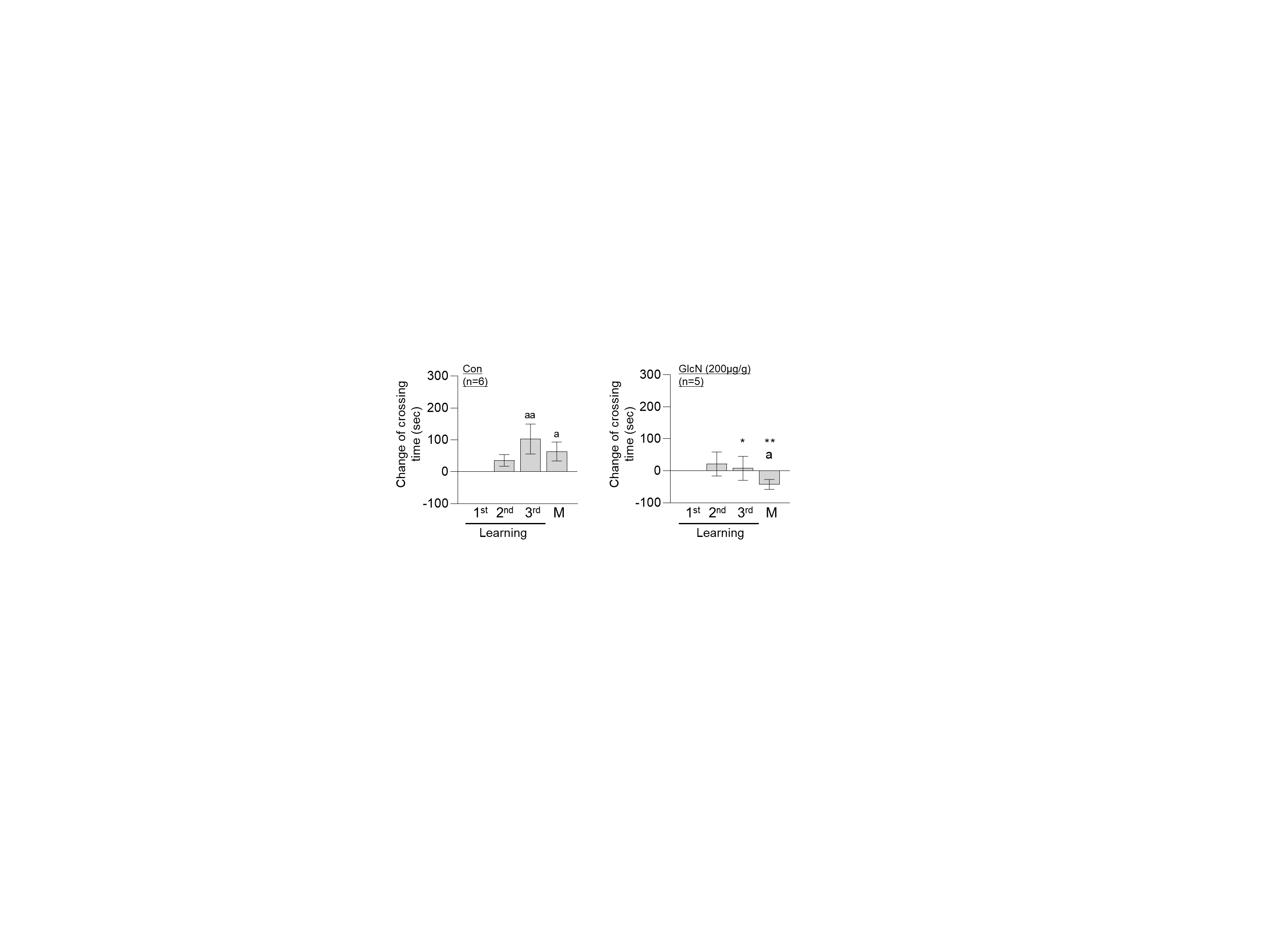

Supplement: Supplementary file 3 — Additional file 3: Figure S3. Repeated GlcN injection with high-dose induces L/M deficit. Zebrafish were intraperitoneally injected with GlcN (200 μg/g) for 3 days and recovered 4 days and this cycle was repeated 5 times. The graphs represent fear context L/M test results. They illustrate the altered crossing times compared to those observed during the first learning session and display the mean ± SEM (n = 5 ~ 6/group). For within-group comparisons, the Friedman ANOVA test with the original FDR correction method by Benjamini and Hochberg was performed for statistical analysis (ap < 0.05, aap < 0.01 versus 1st learning). For between-group comparisons, a two-way ANOVA with Tukey’s multiple comparisons test was performed for statistical analysis (*p < 0.05, **p < 0.01 versus Con). [file 12974_2023_2944_MOESM3_ESM.tif]

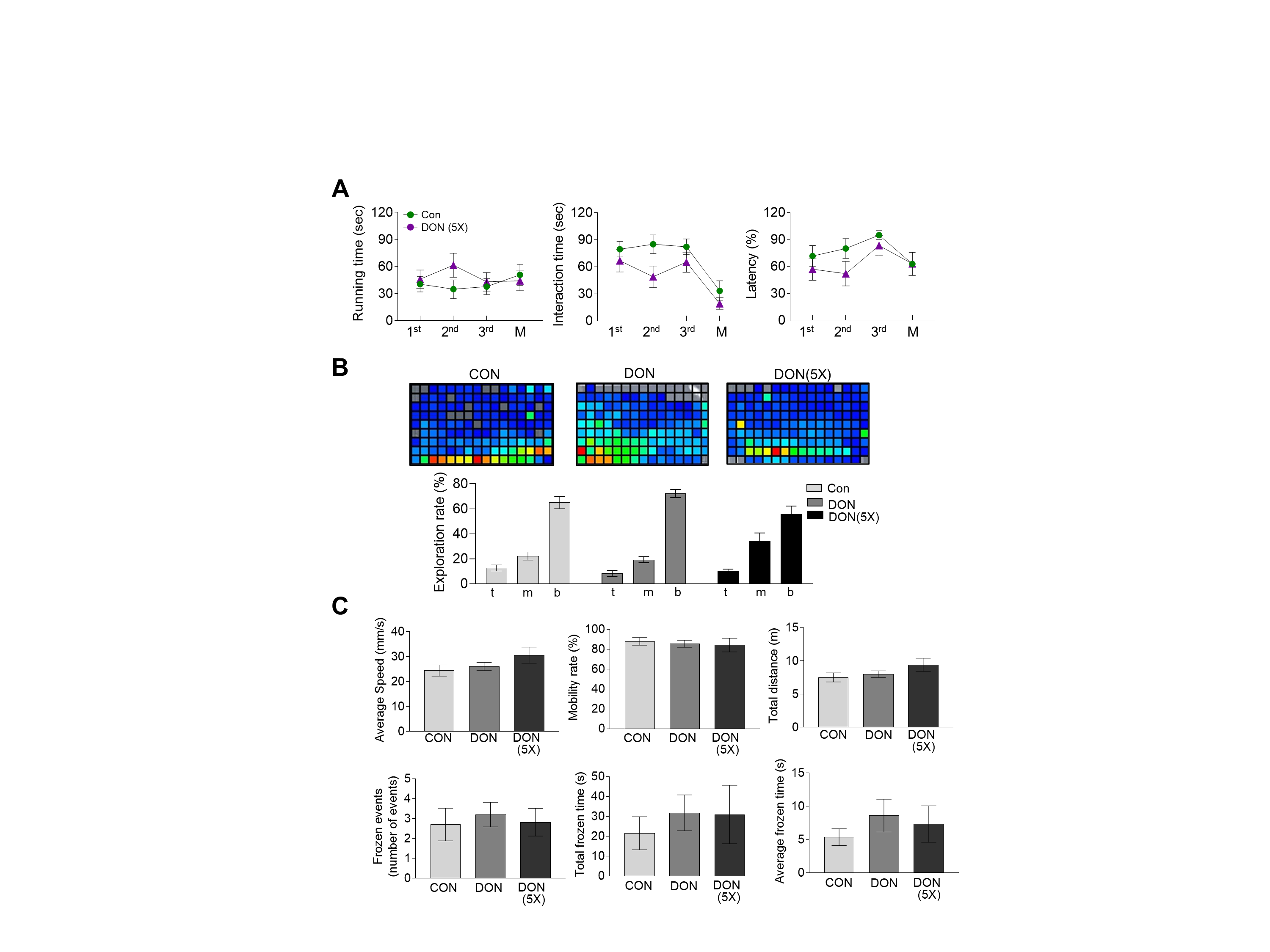

Supplement: Supplementary file 4 — Additional file 4: Figure S4. No changes in social ability and anxiety of the zebrafish after single or repeated DON treatment. Social ability and anxiety in adult zebrafish were assessed following single or repeated cycles of DON injections. DON (500 ng/g) was administered intraperitoneally for 3 consecutive days (DON). For the repeated episodes of DON injection, a cycle of 3 days of injection was followed by 4 days of recovery, and this cycle was repeated 5 times. A day after the final cycle of DON injections, behavior tests were conducted (DON (5X)). (A) Social behavior was assessed using the T-maze test. The graphs display the running time (time taken to reach the friends’ zone), interaction time (duration of interaction near the friends’ zone), and latency (number of trials required to reach the correct arm). Statistical analysis was conducted using a two-way ANOVA with Tukey’s multiple comparisons test (n = 10/group, n.s). (B and C) Anxiety was measured using the NTT test in DON or DON (5X) zebrafish. (B) The heatmap images represent the distribution during novel tank exploration. The graphs represent the tendency of zebrafish exploration in the top (t), middle (m), and bottom (b) of the novel tank with mean ± SEM (n = 10 ~ 15/group). For statistics analysis two-way ANOVA followed by Tukey multiple comparisons test was performed (n.s). (C) Representative graphs demonstrate the average speed, mobility rate, total swim distance, number of freezing events, and average or total freezing time. The data represent the mean ± SEM (n = 10 ~ 15/group). A two-way analysis of variance (ANOVA) was performed followed by Tukey’s multiple comparisons test for statistical analysis (n.s). [file 12974_2023_2944_MOESM4_ESM.tif]

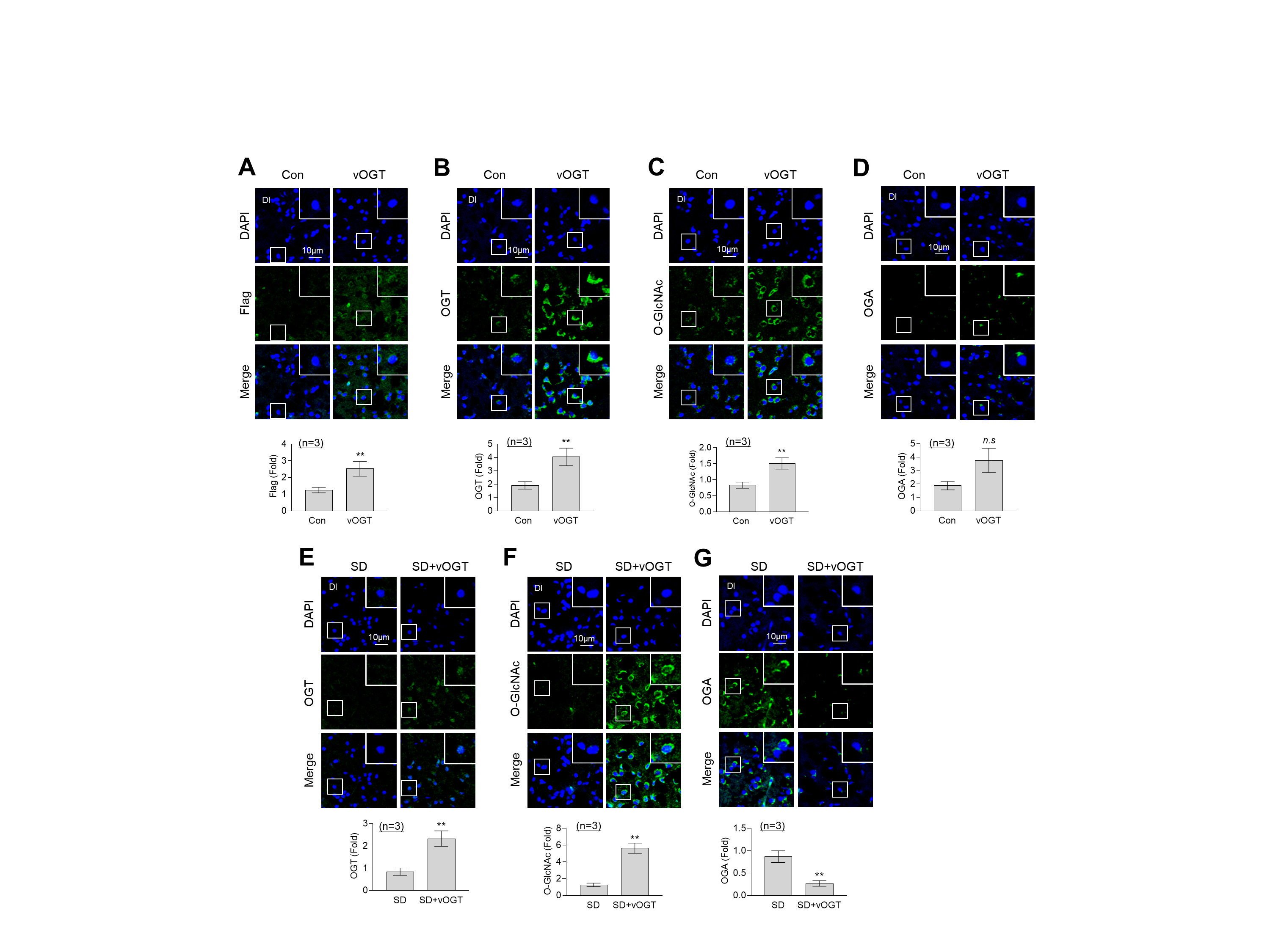

Supplement: Supplementary file 5 — Additional file 5: Figure S5. Changes in OGT, OGA, and O-GlcNAcylation induced by OGT overexpression in the zebrafish brain. AAV8–CMV–mOGT–Myc–Flag (vOGT), a virus containing murine OGT overexpression construct, was injected into the zebrafish brain. Two weeks after the injection, zebrafish brains were sacrificed and analyzed. (A–D) Representative confocal images (40x) of DAPI (blue), Flag (A, green), OGT (B, green), O-GlcNAc (C, green), OGA (D, green), and merged images depict the immunofluorescence staining of the Dl regions of zebrafish telencephalon. Enlarged images are shown in the white boxes. The graphs present the quantitative results for each antibody, with normalization based on the DAPI levels (n = 3/group). For statistical analysis, the Kruskal–Wallis test with the original FDR correction method of Benjamini and Hochberg was performed (**p < 0.01 versus Con). (E–G) Two weeks after the vOGT injection, zebrafish were subjected to SD. Zebrafish brains were then sacrificed and analyzed after the SD. Representative confocal images (× 40) of DAPI (blue), OGT (E, green), O-GlcNAc (F, green), OGA (G, green), and merged are shown. These images depict the immunofluorescence staining of the Dl regions of zebrafish telencephalon. Enlarged images are presented in the white boxes. The graphs represent the quantitative results for each antibody, with the data normalized by the DAPI levels (n = 3/group). For statistical analysis, the Kruskal–Wallis test with the original FDR correction method by Benjamini and Hochberg was performed (**p < 0.01 versus SD). [file 12974_2023_2944_MOESM5_ESM.tif]
